# Hydraulic Signaling in Plants: From Physical Perturbation to Distributed Perception and Context-Dependent Decoding

**DOI:** 10.3390/plants15162513

**Published:** 2026-08-20

**Authors:** Nanyang Li, Wenyuan Wang, Ruichao Li, Binglei Zhang

**Affiliations:** School of Landscape and Ecological Engineering, Hebei University of Engineering, Handan 056038, China; linanyang130430@126.com (N.L.);

**Keywords:** hydraulic signaling, hydromechanics, mechanosensing, osmosensing, aquaporins, Ca^2+^ signaling, systemic acclimation, drought, salinity, wounding

## Abstract

Hydraulic perturbations are among the earliest plant-wide consequences of drought, salinity and wounding, yet they are often treated as passive outcomes rather than as biologically interpreted inputs. This review distinguishes hydraulic state, hydraulic perturbation and hydraulic signal, and evaluates how organ-scale pressure and water-potential changes are converted into local membrane tension, wall strain, turgor and water-flux cues. We propose, as a testable model rather than an established mechanism, a distributed architecture comprising OSCA/TMEM63 and other mechanosensitive channels, cell-wall integrity pathways, aquaporin-mediated conductance control and vacuolar buffering. Evidence for the individual components is substantial, but evidence that they act together within a single physiological event is still limited. These layers are reciprocally coupled to Ca^2+^, ROS, electrical, hormonal and peptide networks. Hydraulic cues are fast, and they differ in amplitude, direction, rise time, duration, recovery and anatomical route, so they are not informationally inert. Specificity nevertheless appears to emerge from the integration of the hydraulic waveform with tissue state and coincident ionic, electrical and biochemical inputs rather than from any single variable. We compare drought, salinity and wounding; clarify the roles of roots, vasculature, bundle sheath, mesophyll and guard cells; and outline experiments that combine calibrated physical perturbations with live reporters, tissue-specific genetics and hydromechanical modeling. The key frontier is no longer to document that pressure changes occur. It is to identify the variables directly sensed, to separate instructive from permissive roles, and to test whether dynamic decoding traits improve crop resilience at acceptable carbon and growth cost.

## 1. Introduction

Plants inhabit environments in which water status, tissue pressure and mechanical load fluctuate continuously. Soil drying raises resistance at the root–soil interface and limits uptake before bulk soil water is exhausted [1]; salinity imposes an abrupt osmotic disequilibrium that lowers root xylem pressure within about a minute of exposure [2]; and wounding breaks pressure continuity and vascular integrity, generating both local deformation and long-distance pressure redistribution [3]. In each case, changes in water potential, pressure, turgor and tissue deformation rank among the earliest plant-wide consequences of stress [4]. These physical events develop within seconds to minutes, and in *Arabidopsis*, a hydraulic component of root-to-shoot communication precedes the ABA-dependent events that complete stomatal regulation in the shoot [5].

Classical plant hydraulics established the soil–plant–atmosphere continuum as a physically coherent transport system, and that framework remains indispensable for interpreting transpiration, growth limitation, leaf water status and stomatal behavior. A strict separation between physics and biology is nevertheless difficult to maintain, and three independent lines of evidence, obtained in different species across three decades, converge on the same conclusion. In wheat, localized burning raises turgor in the epidermis of adjoining leaves, and, decisively, a pressure wave imposed at the tip of one leaf in the complete absence of wounding travels to neighboring leaves and induces an apoplastic potential change resembling a variation potential [6]. In pea, a defined step increase in xylem pressure applied at the root of intact seedlings propagates almost immediately through the epicotyl, decrementing with distance because the xylem is hydraulically leaky, and elicits slow wave potentials in the surrounding living cells [7]. In *Arabidopsis*, long-distance changes in turgor pressure locally activate glutamate receptor-like (GLR) channels in systemic tissue, providing the molecular counterpart to those classical observations [8]. Together they establish that a controlled physical input, delivered without wounding or chemical release, is sufficient to elicit an electrical and ionic response in living cells.

Comparable coupling operates under salinity, where salt addition to potato roots triggers hydraulic and Ca^2+^ waves that reach the shoot before the earliest measurable decline in photosynthetic activity, and pharmacological suppression of the Ca^2+^ component attenuates that early response [9]. Rapid systemic responses to localized water limitation, salinity and tissue damage therefore unfold on time scales consistent with a substantive contribution from physical cues, acting either as signals in their own right or as permissive inputs that shape biochemical signaling [10].

The field is now positioned for a more mechanistic synthesis. Poroelastic modeling yields quantitative predictions for the generation, attenuation and tissue deformation associated with local and long-distance perturbations [3]. DUF221/OSCA proteins were identified as osmosensitive Ca^2+^-permeable cation channels conserved across eukaryotes [11]; OSCA1 was shown to mediate a major component of the hyperosmotic-stress-evoked cytosolic Ca^2+^ increase [12]; and structural and electrophysiological analysis established that several OSCA/TMEM63 members are intrinsically mechanically activated channels [13]. The identification of OSCA2.1 and OSCA2.2 as cell-surface hypo-osmosensors in pollen demonstrates that at least some plant cells monitor extracellular water availability with molecular specificity [14]. In parallel, FERONIA-dependent maintenance of wall integrity under salt stress [15], kinase- and trafficking-based control of aquaporin activity [16,17], and turgor-dependent protection of the tonoplast after wall damage [18] have broadened the candidate sensing architecture well beyond a single receptor class.

This review uses information in a functional biological sense rather than as a formally quantified Shannon metric. A hydraulic perturbation is treated as a signal only when it is sensed, decoded and used to alter physiology in another cellular domain, tissue or organ. That criterion separates biologically interpreted events from background fluctuation in water status, and directs attention to the causal sequence running from signal generation and propagation through local perception to physiological output.

Three propositions organize what follows. First, the organ-scale variables propagated through xylem or surrounding tissues are not the variables that individual cells actually sense. Second, perception is distributed among membrane channels, the cell wall–plasma membrane–cytoskeleton continuum, regulated water permeability and intracellular buffering. Third, hydraulic cues contribute speed and coarse information about magnitude or rate, whereas response specificity emerges from integration with Ca^2+^, ROS, electrical activity, hormones, peptides, tissue identity, developmental state and prior stress exposure.

These propositions also define what this review adds to existing syntheses. Hydromechanical transmission has been formalized as a unified physical theory [3]; osmosensing has been surveyed at molecular and crop scales [19,20]; and rapid systemic signaling has been reviewed from the standpoint of Ca^2+^, ROS and electrical waves [10]. What has not been assembled is a single account that does three things at once. It should separate hydraulic state, perturbation and signal through an explicit perception criterion. It should treat perception as distributed across four physically distinct layers rather than assigning it to one receptor class. And it should convert that architecture into falsifiable predictions with a matched experimental design. Section 2, Section 3, Section 4 and Section 5 build the framework, Section 6 tests it against three stress contexts, and Section 7 and Section 8 specify the experiments capable of refuting it.

## 2. Defining Hydraulic Information in Plants

### 2.1. Hydraulic State, Hydraulic Perturbation and Hydraulic Signal

The phrase hydraulic signal is used inconsistently. Hydraulic state denotes the instantaneous condition of a cell, tissue or plant with respect to water potential, osmotic potential, turgor pressure, xylem tension, hydraulic conductance and related variables. Hydraulic perturbation denotes a change in one or more of these variables, such as the fall in root xylem pressure that follows salt exposure [2], the redistribution of tissue tension after wounding [3], or the progressive decline in leaf turgor during soil drying. Hydraulic signal adds a biological criterion: the perturbation must be perceived and translated into a response at another location or in another signaling layer [21]. Because these terms are frequently conflated, we make the underlying levels of evidence explicit and use them consistently throughout this review. Level 1 establishes only that a physical perturbation occurs, for example, a measured fall in xylem pressure. Level 2 establishes that the perturbation propagates and characterizes its attenuation and filtering. Level 3 establishes local transduction, that is, an electrical, ionic or biochemical response that follows the physical input in the receiving cell. Level 4 establishes that this response alters physiology in a way that has, or plausibly has, an adaptive consequence. Only Level 4 licenses the term hydraulic signal as defined here. The distinction that matters most for the present argument is between Levels 3 and 4. A calibrated pressure step that elicits an electrical response demonstrates that a physical input is sufficient to trigger transduction under controlled conditions; it does not demonstrate that the same perturbation is the endogenous carrier under drought, salinity or wounding, nor that the resulting response is adaptive. Statements in this review are labeled by the highest level they actually reach, and inferences that require Level 4 are identified as proposals wherever the supporting evidence stops at Level 3.

This distinction matters because plants experience routine hydraulic fluctuation that need not trigger acclimation. Diurnal changes in transpiration alter leaf water potential, yet tissues may buffer them without initiating a stress program. Conversely, a short-lived perturbation with an unusually steep onset can be informative if it reliably predicts a developing stress. Signal status therefore depends on amplitude, rate of change, duration, spatial origin, tissue context and decoding competence, not on the existence of a pressure or water-potential change alone.

### 2.2. Temporal and Spatial Scales

Pressure redistribution occurs rapidly through hydraulically connected tissues. In maize roots, salt addition produces a fall in root xylem pressure within about one minute, before Na^+^ is detectable in the xylem vessels [2]. The physical event therefore precedes ion delivery to the shoot rather than reporting it. Section 6.2 describes the experiment in full. In *Arabidopsis*, salt application to roots initiates a Ca^2+^ wave that propagates to the shoot on a comparable time scale [22]. During soil drying, reciprocal grafting showed that a hydraulic component precedes ABA-dependent events in the shoot, while ABA biosynthesis and signaling in the shoot remain necessary for the full stomatal response [5]. Poroelastic modeling places these observations in a fluid–solid framework in which pressure redistribution inside conduits is coupled to deformation of the surrounding living tissue [3].

The kinetics of the physical event have been resolved directly. Displacement transducers ranged along a single wheat leaf show that localized wounding causes a rapid, systemic increase in leaf thickness, and that this thickness change closely tracks leaf water potential [23]. The wound-induced hydraulic signal travels at the order of centimeters per second, faster than any chemical or electrical carrier known in the same tissue. Measured propagation velocities for the downstream biological waves are correspondingly slower, at roughly 8.4 cm per minute for the RBOHD-dependent systemic ROS signal [24]. Expressed in common units, these are approximately 600 cm min^−1^ and 8.4 cm min^−1^, so the two differ by roughly 70-fold rather than by several hundred-fold. Even this smaller separation constrains causal ordering, because a wave arriving more than an order of magnitude later cannot carry the earliest distal event. It is useful to place the other rapid carriers on the same axis. Systemic Ca^2+^ waves triggered by root salt exposure advance at the order of hundreds of micrometers per second [22], which is comparable to the ROS wave and consistent with their mechanistic coupling [25]. Electrical signals span a wider range: action potentials in higher plants typically propagate at the order of millimeters to a few centimeters per second, whereas variation potentials are slower, decrement with distance and vary strongly with stimulus and tissue [23,26,27]. Chemical carriers moving in the transpiration stream are limited by bulk flow and by loading and unloading kinetics, so root-to-shoot delivery of a xylem-borne signal such as abscisic acid operates on a scale of many minutes to hours [5]. Hydraulic propagation is therefore the fastest of these routes by a clear margin, which is what makes its temporal precedence informative, but speed alone does not establish that it carries the instruction.

Wounding has yielded the most complete kinetic description. Localized burning of one wheat leaf raises turgor pressure in epidermal cells of adjoining leaves, and the propagating pressure wave arises from relief of xylem tension by water released from the damaged region [6]. Displacement transducers ranged along a single leaf placed the velocity of the wound-induced hydraulic front at 10 cm s^−1^ or more, with the associated increase in leaf thickness persisting for an hour or longer and recurring after successive wounds [23]. Because the response also occurred in shoots excised from any external water supply, it reflects internal redistribution of water rather than altered uptake [23]. The decisive control was to impose a pressure wave at the tip of one leaf without wounding at all, which induced an apoplastic potential change in neighboring leaves resembling a variation potential [6]. Taken with the pressure-step experiments in pea [7], this establishes that a hydraulic perturbation is sufficient to produce a distal electrical response independently of any damage-released chemistry.

Speed alone does not establish a signal. Propagation is filtered before molecular perception by xylem conductivity, wall elasticity, hydraulic capacitance, vessel geometry, apoplastic barriers and tissue prestress. Radial leakage of water from the xylem into surrounding cells attenuates an applied pressure step as it travels, which is why the electrical response it evokes also decays with distance [7]. These properties determine amplitude, attenuation, dispersion and the identity of the cells that ultimately experience a local mechanical or osmotic change. Biological filtering therefore begins at the level of transport and material properties, before any channel or receptor is engaged.

Four dynamic descriptors are especially useful for comparing hydraulic events: amplitude, rise time, persistence and recovery. Amplitude reflects severity but can be strongly buffered by capacitance; rise time separates abrupt osmotic or wound events from gradual soil drying; persistence distinguishes a transient fluctuation from sustained loss of water supply; and recovery reveals whether the system behaves elastically, acclimates to a new state or approaches failure. Direction also matters, because dehydration and rehydration impose different membrane and wall trajectories. Reporting these four descriptors would make experiments far more comparable than assigning a single label such as drought or osmotic stress.

Hydraulic perturbations span several scales. At the subcellular scale they alter membrane tension, wall strain, cytoplasmic crowding, vacuolar volume and ion-channel activity. At the tissue scale they modify apoplastic continuity, radial conductance and pressure distribution among cell files and veins. At the organ scale they affect xylem pressure, transpiration and root-to-shoot coordination. These scales should not be collapsed. A distal leaf cell does not sense xylem pressure in the abstract; it senses local derivatives of the organ-scale event, including membrane tension, wall deformation, turgor change, water flux or apoplastic pressure.

The same xylem pressure change can therefore be decoded differently in bundle sheath, mesophyll, epidermal and guard cells, because their wall architecture, membrane composition, hydraulic connections and signaling competence differ. Radial root hydraulic conductivity itself varies by an order of magnitude across measurement scales, developmental stages and methods [28], so the perturbation delivered to distal targets is transformed well before it arrives. The route by which water crosses the root is an essential part of this transformation and is treated explicitly here. Radial transport proceeds by three parallel routes arranged in series across successive cell layers: an apoplastic route through the continuous cell-wall space, a symplastic route through plasmodesmata, and a transcellular route across successive membranes in which aquaporins set the resistance. In the composite transport model, the relative contribution of these routes is not fixed. It depends on the driving force and on the developmental state of apoplastic barriers [29]. A hydrostatic gradient generated by high transpiration recruits the low-resistance apoplastic route. An osmotic gradient under low transpiration is carried mainly by the cell-to-cell routes, in which aquaporin regulation becomes rate-limiting. Casparian bands and suberin lamellae in the endodermis and exodermis restrict but do not abolish apoplastic flow, and their extent varies with root zone, age and stress history [30,31]. This matters directly for hydraulic decoding rather than only for water balance. Which route carries the perturbation determines which cells experience it. An apoplastically transmitted pressure change loads the wall and the outer face of the plasma membrane of every cell it passes. A transcellular route imposes repeated membrane crossings instead, and delivers the perturbation preferentially to cells with high aquaporin activity. The same organ-scale event therefore reaches a different population of candidate sensors depending on route, and because route allocation is itself regulated by transpiration and by barrier development, the mapping between perturbation and perceiving cell is dynamic. Experiments that manipulate apoplastic continuity, for example by comparing root zones that differ in suberization or by pharmacologically blocking the bypass, while imaging Ca^2+^ or membrane voltage, would test this directly and have not been performed.

### 2.3. Hydraulic Versus Osmotic Information

Hydraulic and osmotic signaling are closely related but not synonymous. Recent syntheses of osmosensing emphasize that plants probably decode a mixture of changes in turgor, membrane tension, fluid volume and water potential rather than any single scalar variable [19,20]. Osmotic stress changes water potential by altering solute concentration, which frequently produces hydraulic consequences including altered turgor, water flux and membrane tension. Not every hydraulic perturbation is primarily osmotic, however. Wounding, tissue compression and abrupt changes in transpirational demand create hydromechanical disturbance even without strong external osmotic shifts [3]. It is therefore more useful to treat the system as a coupled hydromechanical continuum than as a rigid dichotomy. In real tissue, extracellular osmolarity, membrane tension, wall strain, turgor and xylem pressure often change together, yet the variable first sensed by a particular molecular module may differ among contexts.

OSCA biology illustrates the overlap without eliminating the distinction. DUF221/OSCA proteins were characterized as osmosensitive Ca^2+^-permeable channels [11], and several family members are intrinsically mechanically activated [13], consistent with osmotic stress being transduced partly through membrane mechanics. The identification of OSCA2.1 and OSCA2.2 as cell-surface hypo-osmosensors in pollen indicates that extracellular water availability can be decoded with greater specificity than a generic membrane-stretch model predicts [14]. Osmotic and mechanical variables are thus partially separable, but they are frequently read by overlapping modules.

### 2.4. Information Content, Specificity and a Working Definition

Hydraulic cues are analog rather than digital. They vary continuously in amplitude, rate of onset, duration, recovery kinetics and spatial origin. A modest decline in turgor can accompany normal midday transpiration, incipient drought, mild salinity or local injury, yet these conditions require different responses. The central problem is therefore not detection but discrimination.

Combinatorial decoding offers the most plausible solution, provided it is not reduced to a strict division of labor in which hydraulics is nonspecific and chemistry is specific. Hydraulic perturbations are themselves multidimensional. They differ in amplitude, in the sign of the change, in rise time, in duration, in recovery kinetics, in spatial origin and in the anatomical route by which they travel, and these properties are measurable and physiologically consequential. A slow decline in leaf turgor during soil drying and an abrupt depressurization after stem wounding are not the same physical event, and there is no reason in principle why a decoding system should be blind to that difference. What the available evidence does not support is the stronger claim that any single hydraulic variable uniquely identifies a stress. The defensible position is intermediate: the hydraulic waveform carries genuine information about magnitude, rate and location, while ionic, biochemical and electrophysiological pathways supply additional, partly orthogonal information, and identity emerges from the joint interpretation of both together with tissue state and anatomical context. Similar perturbations produce different outcomes depending on whether they coincide with sodium influx and membrane depolarization [2], with wound-associated glutamate and GLR-dependent electrical activity [32,33], or with a ROS-assisted Ca^2+^ wave requiring RBOHD and TPC1 [25].

A candidate molecular implementation of coincidence detection has been proposed, although its status should be stated carefully. The stretch-activated anion channel MSL10 is required for normal wound-induced electrical and Ca^2+^ signaling in distal leaves and acts within the same genetic pathway as the glutamate receptor-like proteins [34]. The interpretation that GLRs operate as coincidence detectors gated by the joint requirement for ligand binding and membrane depolarization, with the depolarization supplied by stretch activation of MSL10, is a model inferred from genetic epistasis and from the known properties of the two channel classes. Direct biophysical demonstration that a single GLR channel requires the simultaneous arrival of a mechanical and a chemical input has not been reported, and the epistasis is equally compatible with sequential rather than strictly coincident action. This arrangement is formally analogous to the dual ligand and voltage requirement of mammalian NMDA receptors, and it provides a concrete molecular route by which a mechanical variable and a chemical variable must arrive together before a distal response is licensed. It also predicts that a hydraulic perturbation delivered without the accompanying chemical cue, or the reverse, should be comparatively ineffective, which is a directly testable claim.

**Figure 1:**
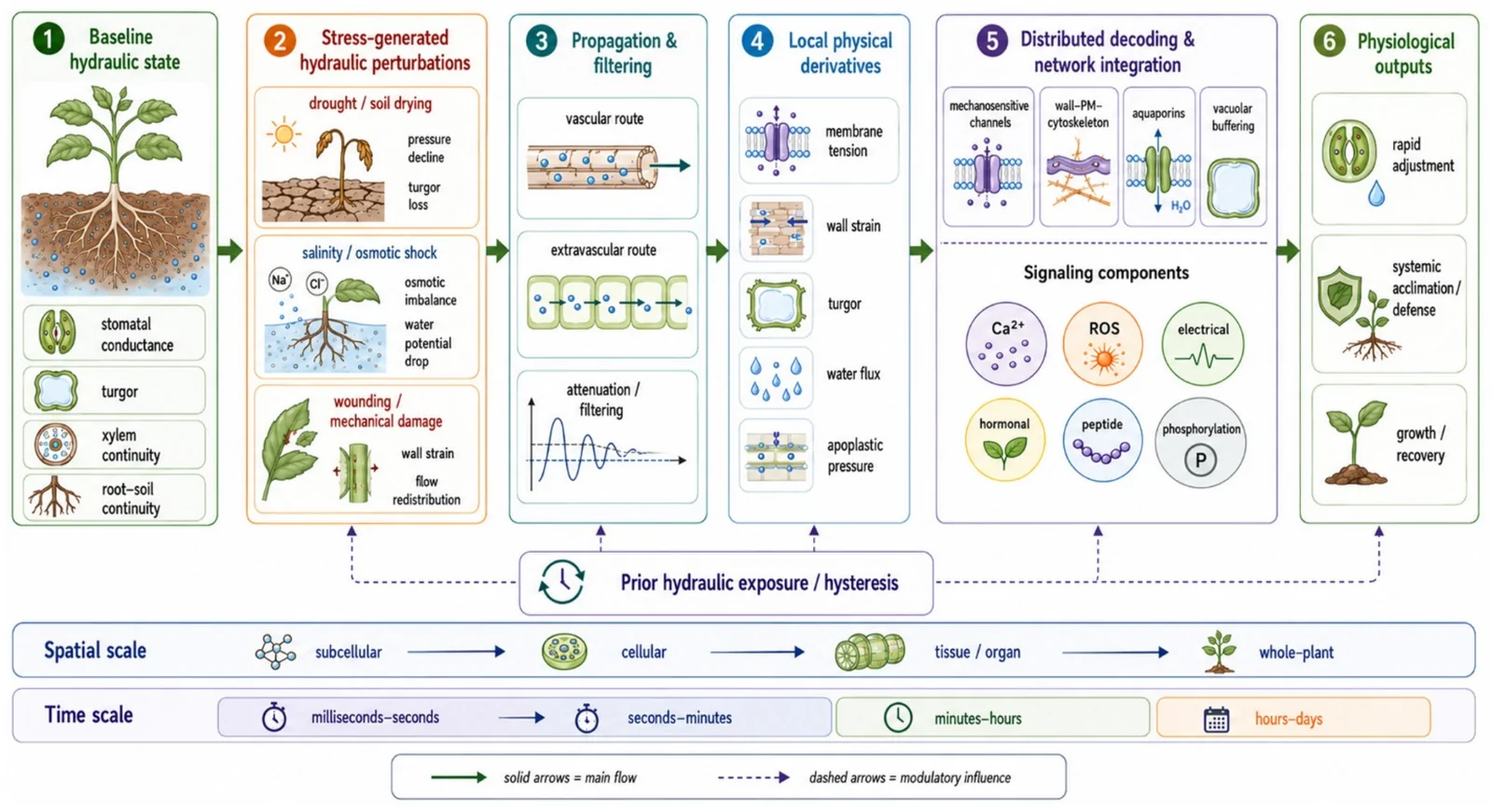
A multiscale framework for the generation, propagation, perception and physiological integration of hydraulic perturbations in plants. (1) The baseline hydraulic state is defined by stomatal conductance, cell turgor, xylem continuity and root–soil continuity. (2) Drought, salinity and wounding generate hydraulic perturbations that differ in onset kinetics and in accompanying chemical or ionic context rather than in the physical variables they engage. (3) Perturbations travel by vascular and extravascular routes and are attenuated and filtered by conduit geometry, hydraulic capacitance, apoplastic barriers and tissue prestress before any receptor is engaged. (4) What reaches a distal cell is therefore a set of local physical derivatives, namely membrane tension, wall strain, turgor change, water flux and apoplastic pressure, rather than the organ-scale variable itself. (5) These derivatives are decoded by four functionally distinguishable layers comprising mechanosensitive and osmosensitive channels, the cell wall–plasma membrane (PM)–cytoskeleton continuum, aquaporins, and vacuolar buffering, which are reciprocally coupled to Ca^2+^, reactive oxygen species (ROS), electrical, hormonal, peptide and phosphorylation-based signaling. Phosphorylation is shown among the signaling components rather than downstream of them, because subclass I SnRK2 kinases are activated by hyperosmotic stress on a time scale comparable to the ionic and electrical layers and independently of abscisic acid (Section 3.3). (6) Context-dependent physiological outputs follow. A perturbation qualifies as a hydraulic signal only where stages 4, 5 and 6 all occur, that is, only where it is locally perceived, decoded and used to alter physiology elsewhere. This matches the textual definition in Section 2.1, in which a physiological output is required, and local transduction alone is insufficient. Prior hydraulic exposure is drawn as a modulatory input to stages 2 to 6. This is hydraulic hysteresis in the material sense: a biophysical precedent is documented at the tissue scale, whereas an active cellular mechanism and a demonstrated functional consequence are both lacking, so the arrangement should not be read as biological memory. Solid arrows denote the main flow of information between stages and dashed arrows denote modulatory influence. Within the stage 5 module icons, the double-headed blue arrow crossing the lipid bilayer of the aquaporin denotes water flux; it is drawn double-headed because the direction of flux is set by the sign of the local water-potential gradient and is not fixed. Aquaporins and mechanosensitive channels are drawn as integral proteins spanning a bilayer, consistent with their function as membrane transport proteins. Spatial and temporal scales are indicative and are not drawn to scale.

Accordingly, hydraulic signaling is defined here as the generation, transmission, perception and biological interpretation of stress-induced changes in water-related physical variables, including water potential, turgor, xylem tension, tissue pressure and the associated deformation, that are used by cells, tissues or organs to adjust physiology during acclimation. This conservative definition excludes unperceived background fluctuation, assumes no universal receptor, and separates organ-scale propagation from the local variables that living cells actually decode (Figure 1).

## 3. A Distributed Architecture for Hydraulic Sensing and Decoding

### 3.1. Plasma-Membrane Mechanosensors and Osmosensors

The plasma membrane is the most immediate interface at which a hydraulic perturbation can become ion flux. Changes in turgor and water movement alter membrane tension, curvature, lipid organization and membrane–wall coupling within milliseconds to seconds, and these properties change the open probability of mechanically sensitive channels [13]. The strongest candidates are OSCA/TMEM63 proteins, MSL channels, MCA proteins and PIEZO. For each of them, the evidence for channel mechanosensitivity is substantially stronger than the evidence for a direct role in decoding long-distance hydraulic signals [35].

OSCA1 is required for a major component of the hyperosmotic-stress-evoked cytosolic Ca^2+^ increase in *Arabidopsis* [12], and structural and electrophysiological analysis shows that several OSCA/TMEM63 proteins are mechanically activated channels [13]. Together these findings establish a credible route from membrane mechanics to ionic signaling. They do not demonstrate that all OSCAs are generic hydraulic receptors, nor that any single family member senses a whole-plant pressure wave. Tissue expression, developmental state, membrane lipid composition, phosphorylation and wall attachment are all likely to determine whether a given OSCA acts as a primary transducer, a downstream amplifier or neither.

Structural work has since specified how a mechanical input becomes conductance, which is the step most often left as a black box. Cryo-electron microscopy showed that OSCA1.2 is a dimer carrying eleven transmembrane helices per subunit, with an unexpected topological resemblance to TMEM16 lipid scramblases and with molecular-dynamics evidence that membrane lipids participate in gating [36]. A subsequent set of 44 structures, determined across different membrane environments, captured the gating cycle itself [37]. In nanodiscs under conditions mimicking raised membrane tension, one subunit displayed a dilated pore open laterally to the membrane. In liposomes the fully open conformation showed a large lateral opening. Lipids therefore form part of the wall of the ion permeation pathway. In the less tension-sensitive homologue OSCA3.1, an interlocking lipid bound in the central cleft holds the channel closed, and mutating the residues that coordinate that lipid is sufficient to open it; each subunit gates independently [37]. Membrane tension is therefore no longer a phenomenological input but a defined structural transition, and the involvement of bound lipids predicts that membrane composition should itself modulate hydraulic sensitivity. It is worth setting out how such gating is thought to work, because the step from a tissue-scale pressure change to an open channel is where hydraulic signaling is most often left underspecified. Two non-exclusive paradigms are available. Under the force-from-lipids principle, the bilayer is the terminal transducer. In-plane tension, bilayer thinning under stretch, hydrophobic mismatch between channel and surrounding lipid, and local curvature together shift the energetic balance between the closed and open states. A channel reconstituted in pure liposomes therefore remains mechanosensitive with no other cellular component present [38]. Under the force-from-filament paradigm, force reaches the channel through tethers to the wall or the cytoskeleton, and the channel reports relative displacement rather than bilayer tension as such. Plant plasma-membrane channels are embedded in a wall–membrane–cytoskeleton continuum [39], so both routes are available in principle, and distinguishing them experimentally requires that wall attachment and cytoskeletal integrity be manipulated independently of tension. Structural work on OSCA supports a substantial force-from-lipids contribution, since lipids occupy the permeation pathway and an interlocking lipid holds the low-sensitivity homologue closed [37], and structures of MSL10 in open and closed states similarly place the gating transition within the membrane [40].

This framework makes membrane lipid composition a direct determinant of channel activity rather than a passive background, which is the second half of the question. Reconstitution experiments with the bacterial channels MscL and MscS remain the reference system for bilayer-mediated gating, and they show that the tension threshold for opening depends on the lipid environment [41]. Lyso-lipids inserted asymmetrically into one leaflet alter the transbilayer pressure profile and shift activation. Raising the degree of acyl-chain unsaturation changes bilayer stiffness and thickness, and so changes how efficiently membrane tension reaches the channel. Bilayer thickness, sterol content, the abundance of anionic phospholipids and phosphoinositides, and local curvature all enter the same energetic balance [38]. The functional prediction for plants is direct and testable: because membrane lipid composition differs among tissues, among developmental stages and with prior stress exposure, the same organ-scale pressure change should produce different channel activity in cells that differ in lipid environment. This offers a mechanistic route to tissue-specific decoding thresholds that does not require differences in channel abundance, and it has not been tested in any plant hydraulic system. Channel activity is set further by post-translational regulation and by inactivation kinetics, so a sustained perturbation need not produce a sustained current. Adaptation and desensitization at the channel level would act as a high-pass filter that favors rapid-onset events over slow ones. Waveform shape, and not amplitude alone, would then become biologically consequential. Pollen provides the clearest demonstration of specialized water-status sensing. Reduced extracellular osmolarity enhances Ca^2+^ oscillations during germination, and osca2.1 and osca2.2 pollen is defective in this response [14]. The result should not be extrapolated uncritically to vegetative tissue, because pollen has unusual wall mechanics and undergoes extreme rehydration. It nevertheless shows that hyperosmotic dehydration and hypo-osmotic rehydration need not be mirror images decoded solely by generic stretch.

Other channel families broaden the candidate landscape. MSL9 and MSL10 together account for the major mechanosensitive channel activity of *Arabidopsis* root protoplasts [42], whereas the pollen-specific MSL8 acts as a tension-gated osmotic safety valve during hydration and germination [43]. The open conformation of MSL10 has now been resolved, which defines how membrane tension couples to the conducting state [40]. MCA1 is required for mechanically induced Ca^2+^ influx and touch sensing in roots [44], and *Arabidopsis* PIEZO is required for mechanically evoked root-tip Ca^2+^ transients and for penetration of dense media [45]. For most of these families the evidence remains local, but MSL10 is a documented exception. Mutant *msl10* plants show markedly shortened wound-induced slow wave potentials in distal, unwounded leaves, and this defect is not additive with loss of GLR3.3 or GLR3.6 [34]. A stretch-activated channel therefore lies within the same long-distance pathway as the glutamate receptor-like channels. A mechanosensitive channel therefore does contribute to systemic signaling, at least in the wound context.

The clearest available demonstration that a hydraulic variable is decoded, rather than merely transmitted, comes from work separating the propagating variable from the perceived one. Wounding, burning and hypo-osmotic treatment of roots each raise apoplastic L-glutamate in distant tissue, and this systemic rise occurs largely independently of GLR3.3; GLR3.3 is instead required for the subsequent cytosolic Ca^2+^ elevation, and its activation depends on a functional ligand-binding domain. Critically, releasing comparable amounts of L-glutamate locally in the leaf lamina fails to generate any long-distance Ca^2+^ wave, which excludes glutamate itself as the mobile carrier and identifies long-distance turgor pressure change as the propagating variable that licenses local channel activation in systemic tissue [8]. This result supplies the mechanistic template that the present framework requires: an organ-scale physical variable travels, a local chemical and receptor step confers specificity, and the two are experimentally separable.

A useful experimental hierarchy separates channel biophysics from signaling function. Heterologous expression or patch-clamp recording can establish mechanical gating; loss-of-function phenotypes can establish physiological necessity in a defined tissue; and tissue-specific rescue can identify the relevant anatomical site. A bona fide hydraulic-decoding mechanism additionally requires evidence that a calibrated hydraulic event reaches that site, activates the channel at the appropriate latency, and changes a distal response without merely altering development or basal water transport. Applying this hierarchy would prevent mechanosensitivity from being equated automatically with whole-plant hydraulic sensing.

### 3.2. The Cell Wall–Plasma Membrane–Cytoskeleton Continuum

Hydraulic perturbations do not act on isolated membranes. The wall constrains expansion, the membrane receives and transduces force, and the cytoskeleton reorganizes transport, wall deposition and cell shape. Force is distributed across a wall–membrane–cytoskeleton continuum rather than delivered to a single freely suspended channel [39,46].

Wall hydration, stiffness, prestress and anisotropy govern how a change in turgor is converted into local strain. Under drought or salinity, wall polymers can stiffen, loosen or redistribute load, whereas wounding creates rupture and abrupt anisotropy. These changes alter membrane–wall attachment, receptor organization and cytoskeletal orientation.

Three lines of genetic evidence establish that plants monitor this mechanical state through dedicated receptors rather than inferring it indirectly. First, THESEUS1, a malectin-domain receptor-like kinase, mediates the cellular response to inhibition of cellulose synthesis [47]. The mutant has no phenotype in an otherwise intact wall and becomes informative only once wall integrity is compromised. That is the signature expected of a damage sensor rather than of a constitutive growth regulator. Second, FERONIA is required to maintain wall integrity specifically under salt stress, acting through cell-type-specific Ca^2+^ transients [15]. Conditions that restore pectin cross-linking rescue *fer* wall defects, which ties the receptor to the mechanical state of the matrix rather than to ionic stress as such. Third, the cell-wall integrity and pattern-triggered immunity systems are not parallel but interdependent, and their cooperation determines the magnitude of the downstream stress response [48]. Wall surveillance is therefore an established signaling layer, even though its direct interrogation in long-distance hydraulic signaling remains scarce [35]. An important qualification applies to all three lines of evidence. They establish that THESEUS1 and FERONIA are required for normal cell-wall integrity signaling, and that this requirement becomes visible when the wall is perturbed. They do not establish that either receptor directly senses the relevant mechanical variable. The proximate stimulus could be wall strain, a change in pectin cross-linking, displacement of a ligand such as a RALF peptide, altered receptor clustering, or a secondary consequence of any of these, and current genetics cannot distinguish among them. Describing FERONIA and THESEUS1 as wall-integrity sensors is therefore a statement about pathway requirement rather than about the physical variable transduced, and the distinction matters for hydraulic decoding because the candidate variables differ in how they would scale with a propagated pressure change.

Wall sensing also reaches inward to the compartment that generates turgor. FERONIA, acting together with Leucine-Rich Repeat Extensins (LRX), senses the extracellular matrix and controls vacuolar expansion during cell elongation, so that a perturbation registered at the wall is translated into a change in the volume of the organelle that sets cellular water status [49]. This finding links the wall layer of this section to the vacuolar layer of Section 3.4 within a single genetic pathway and argues that the layers of the proposed architecture are coupled rather than merely coexisting.

Cell-wall integrity pathways thus provide the route by which a broad physical variable acquires biological meaning. Wall fragments, pectin status, RALF peptides and receptor-like kinases can distinguish reversible deformation from damage that threatens integrity. Outputs include Ca^2+^ entry, ROP signaling, RBOH-dependent ROS, altered proton-pump activity, cytoskeletal reorganization and wall repair [15,48]. Because these same pathways regulate growth [47,49], their activation threshold is probably set by developmental state and tissue prestress. Direct experiments should therefore compare equal wall strain in expanding and in mature tissue rather than infer a universal response from a single organ.

This architecture contributes to specificity. Guard-cell walls, root cortical walls, expanding mesophyll and mature xylem-adjacent cells differ in composition, prestress and receptor complement, so comparable pressure changes need not produce comparable deformation or signaling. The cytoskeleton adds feedback by altering wall deposition, vesicle trafficking, channel organization and mechanical anisotropy, and is best treated as a force-transmission and context-setting layer rather than as a stand-alone hydraulic receptor. The regulatory mechanisms involved deserve to be stated explicitly, because the cytoskeleton is frequently invoked in hydraulic contexts without a stated mechanism. The best-characterized behavior is that of cortical microtubules, which reorient along the direction of maximal tensile stress in the plane of the tissue. In the *Arabidopsis* shoot apex, microtubule arrays align with predicted principal stress directions, and mechanical perturbation of the tissue reorganizes those arrays, establishing a feedback loop between tissue geometry, stress pattern and microtubule-dependent wall anisotropy [50]. Controlled compression of tissue produces quantitatively comparable reorientation, which shows that the response is to imposed mechanical load rather than to growth alone [51]. Because microtubules guide cellulose synthase trajectories, reorientation feeds back onto wall anisotropy and therefore onto how a subsequent turgor change is converted into strain. The actin cytoskeleton contributes on a different time scale, through Ca^2+^-dependent actin-binding proteins that sever, bundle and nucleate filaments, and through the control of vesicle trafficking that determines the surface abundance of aquaporins and of channels themselves. The connection to hydraulic waves specifically, however, must be stated as a gap rather than as a result. The cited work uses imposed compression, ablation and growth-generated stress, not propagated pressure transients, and no study has imaged cytoskeletal reorganization in a distal cell during the passage of a calibrated hydraulic front yet. The mechanistic link is therefore plausible on biophysical grounds and supported for mechanical stress in general, but for hydraulic signaling it remains an inference by analogy.

### 3.3. Aquaporins as Regulated Conductance and Gain-Control Elements

Phosphorylation deserves separate emphasis here because it is the fastest covalent mechanism available for adjusting hydraulic properties, and it is not confined to the ABA pathway. Alongside the ABA-activated subclass III enzymes, plants possess subclass I SnRK2 kinases that are activated within minutes by hyperosmotic and saline stress and that are not activated by ABA at all [52]. Their upstream activators have been identified as B4 Raf-like MAP kinase kinase kinases, which are themselves phosphorylated early in the osmotic stress response and which phosphorylate and activate subclass I SnRK2s in vivo [53]. This matters for hydraulic signaling for two reasons. First, it establishes a phosphorylation route whose kinetics are compatible with the seconds-to-minutes window in which hydraulic perturbations propagate, and which does not wait for hormone biosynthesis, transport and perception. Second, it means that the phosphorylation events observed after an osmotic or hydraulic perturbation cannot be assigned to ABA signaling by default. Whether the ABA-independent branch is activated by membrane tension, by cell volume change, by macromolecular crowding or by a distinct osmosensor is unresolved, and this is precisely the kind of question that calibrated physical perturbation combined with phosphoproteomics is suited to answer. Phosphorylation is treated in this review as a signaling class in its own right, coordinated with Ca^2+^, ROS, and electrical activity rather than downstream of them, and it is represented as such in Table 1 and Table 2. Aquaporins determine how rapidly and how strongly a cell experiences a hydraulic perturbation. High membrane water permeability accelerates equilibration, whereas reduced permeability prolongs transient gradients in turgor or membrane tension. Aquaporins are consequently better described as dynamically regulated conductance elements that tune signal gain, duration and spatial reach than as primary hydraulic receptors [54,55].

The regulatory repertoire is fast and post-translational. The ABA-activated kinase OST1/SnRK2.6 phosphorylates PIP2;1 at Ser121, which raises guard-cell osmotic water permeability and is required for ABA-triggered stomatal closure; a phosphomimetic form restores closure in *pip2;1* plants, whereas a phosphodeficient form does not [16]. Aquaporins additionally conduct H_2_O_2_ into guard cells, linking water permeability to redox signaling during ABA- and pathogen-triggered closure [56]. In roots, salt and salicylic acid downregulate hydraulic conductivity through ROS-activated internalization of PIP aquaporins, and exogenous H_2_O_2_ alone reduces root hydraulic conductivity by up to 90% in under 15 min [17]. Such kinetics place aquaporin regulation firmly within the time window of the physical perturbation itself rather than downstream of transcription.

These controls modify root hydraulic conductivity [57], bundle-sheath-to-mesophyll water transfer [58,59], leaf hydraulics, and stomatal behavior under drought and salinity [60]. ABA-dependent regulation of aquaporins illustrates the reciprocity directly: the hormone alters conductance [61,62], and the altered conductance changes the physical perturbation subsequently encountered by downstream sensors.

Evidence that aquaporins are indispensable regulators of plant water transport is strong [63]; evidence that they directly detect hydraulic information is weak. Reduced aquaporin activity may preserve or amplify a transient pressure difference, whereas increased activity may buffer it. Either outcome changes the input delivered to mechanosensors, wall-associated systems and guard-cell signaling. Aquaporins therefore occupy a central feedback position between transport and decoding without needing to be designated receptors.

The sign of an aquaporin effect cannot be predicted from expression level alone. Increased permeability can improve water supply under one gradient but accelerate dehydration or dissipate an informative transient under another. Subcellular localization, PIP1–PIP2 interaction, tissue hydraulic resistance and the direction of water flow all matter, and the sequence of stomatal closure, embolism and aquaporin gene expression during drought differs among species [64]. Time-resolved measurement of phosphorylation, internalization and membrane permeability is therefore far more informative than endpoint transcript abundance when aquaporins are interpreted as components of a decoding network.

### 3.4. Vacuolar Buffering and Intracellular Responses to Hydromechanical Imbalance

The vacuole contributes most of the volume and turgor of many plant cells and is central to osmotic buffering, ion storage and survival. Changes in vacuolar volume and tonoplast load alter cytoplasmic mechanics and the response to external perturbation. Cell-wall damage activates a turgor-dependent tonoplast ATG8ylation pathway that protects vacuolar integrity, and disruption of this pathway increases vacuolar failure and cell death [18]. Notably, the response is triggered by the mechanical consequence of wall damage rather than by wall chemistry alone, which makes it one of the few characterized intracellular pathways with a demonstrated turgor dependence.

The tonoplast also carries the machinery to convert that mechanical state into an ionic signal. TPC1 encodes the slow vacuolar channel, a Ca^2+^-dependent Ca^2+^-release conductance that dominates vacuolar current at micromolar cytosolic Ca^2+^ and whose loss alters germination and stomatal responses [65]. Its relevance to the present argument is that TPC1 is required for normal propagation of the salt-triggered systemic Ca^2+^ wave [22]. Modeling combined with imaging indicates that vacuolar release through this channel must operate together with apoplastic ROS production if the observed wave velocity is to be reproduced [25]. The vacuole is therefore not only a passive reservoir but a participant in long-distance signal relay. The qualification is that TPC1 involvement in guard-cell Ca^2+^ responses to hormones has been contested, so its contribution is better described as context-dependent than as universal [65].

A further connection runs in the opposite direction, from the wall to the vacuole. FERONIA and Leucine-Rich Repeat Extensins together control vacuolar expansion in elongating cells on the basis of extracellular matrix state [49], which means that the organelle setting cellular turgor is itself under the control of the layer that senses wall mechanics. Wall status, vacuolar volume and turgor consequently form a closed regulatory loop rather than a linear chain, and a perturbation entering at any point can propagate around it.

Taken together, this evidence places the vacuole on the intracellular read-out, relay and execution side of hydromechanical decoding rather than establishing it as a primary sensor. Pressure imbalance activates organellar membrane protection, trafficking and homeostatic repair [18]; tonoplast conductance shapes the ionic signal that results [25,65]; and wall-derived signals set vacuolar volume [49]. Related effects on cytoplasmic crowding, phase behavior and membrane contact sites remain plausible but sparsely tested in plants. Separating buffering from perception will require perturbations that independently manipulate tonoplast mechanics, cellular turgor and plasma-membrane signaling. Two clarifications are needed before this layer can be assigned a role. The first concerns the mechanisms that actually set vacuolar volume, since the vacuole occupies most of the cell and its volume is the dominant determinant of turgor. Volume is set by the osmotic content of the lumen and by the mechanical constraint imposed by the tonoplast and the surrounding wall. Solute accumulation is driven by the proton-motive force generated by the vacuolar H^+^-ATPase and H^+^-pyrophosphatase. That force energizes secondary transport of K^+^, Na^+^, Cl^−^, malate and sugars across the tonoplast. Water then follows through tonoplast intrinsic proteins, whose high permeability keeps cytosol and lumen close to osmotic equilibrium on a subsecond scale. Because tonoplast water permeability is high, a hydraulic perturbation delivered to the cell is partitioned between cytosol and vacuole almost immediately, and the vacuole therefore acts as the principal capacitance element within the cell. Volume is additionally regulated over longer intervals by membrane delivery and retrieval, by fusion and fission of the vacuolar compartment, and by wall-derived constraints, as shown by the FERONIA–LRX pathway that couples extracellular matrix status to vacuolar expansion [49]. The link to hydraulic waves is therefore mechanistically explicit at the level of capacitance and osmotic adjustment, but speculative at the level of instruction: whether a change in vacuolar volume is read as information, rather than simply absorbed, has not been tested.

**Figure 2:**
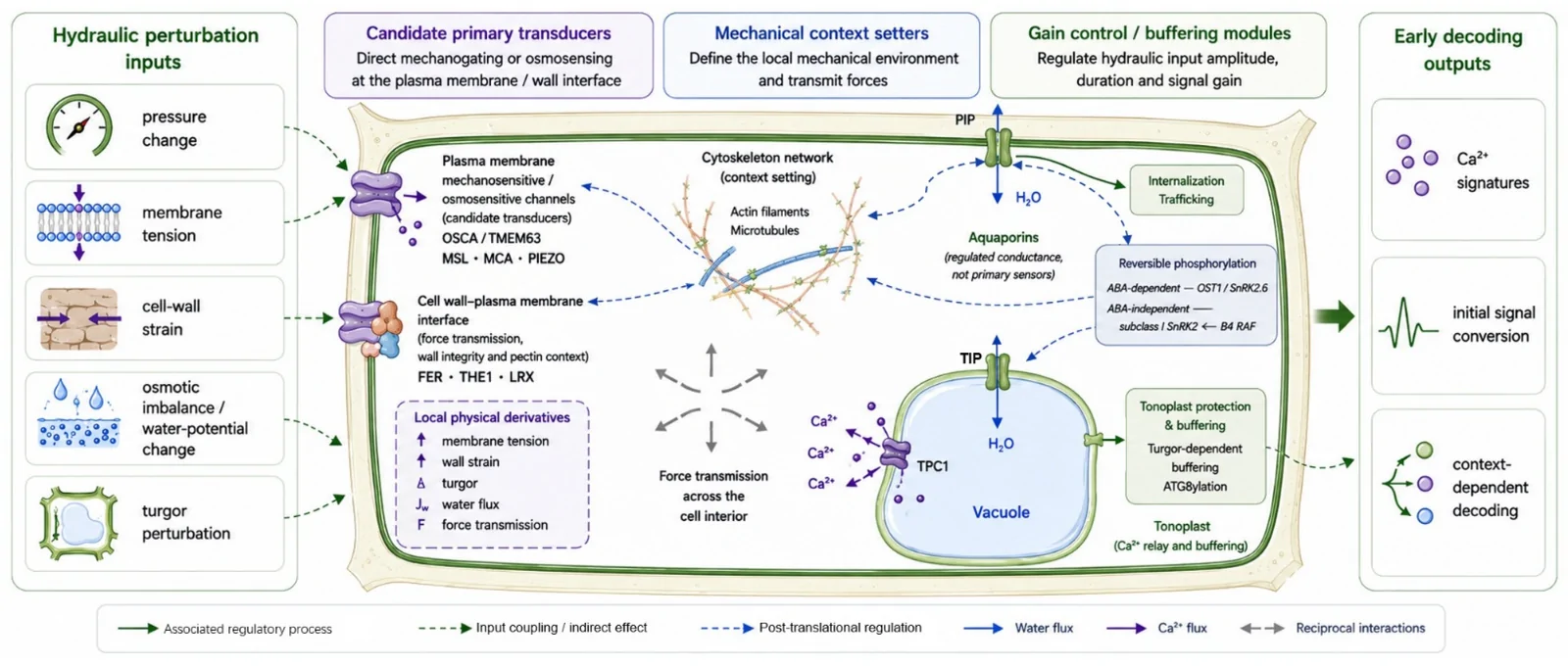
Distributed architecture for decoding hydraulic perturbations within a single plant cell. Hydraulic inputs (left) comprise pressure change, membrane tension, cell-wall strain, osmotic or water-potential change, and turgor perturbation. Three functional categories are distinguished within the cell. Candidate primary transducers mechanogate or osmosense directly at the plasma membrane and wall interface, and include OSCA/TMEM63, MSL, MCA and PIEZO channels. Mechanical context setters comprise two elements. The first is the cell wall–plasma membrane interface, at which FERONIA (FER), THESEUS1 (THE1) and Leucine-Rich Repeat Extensins (LRX) are required for wall-integrity signaling and report on pectin status. The second is the cytoskeleton, drawn as actin filaments and microtubules, which transmits force across the cell interior. Gain control and buffering modules comprise aquaporins and the tonoplast. Aquaporins are drawn as integral channels spanning the lipid bilayer, with plasma-membrane PIPs in the plasma membrane and tonoplast TIPs in the tonoplast, consistent with their role as membrane water-transport proteins. Phosphorylation, internalization and trafficking regulate their permeability. Reversible phosphorylation is shown as a distinct regulatory layer acting on channels, aquaporins and downstream targets, and comprises both ABA-dependent and ABA-independent branches. At the tonoplast, the vacuolar channel TPC1 relays Ca^2+^ in a context-dependent manner and turgor-dependent ATG8ylation protects vacuolar integrity. Local physical derivatives are boxed and comprise increased membrane tension, increased wall strain, change in turgor (Δ), water flux (J_w_) and force transmission (F). Early decoding outputs (right) are Ca^2+^ signatures, initial signal conversion and context-dependent decoding. Aquaporins are positioned as regulated conductance elements that set signal gain and duration rather than as primary sensors. Both water-flux arrows are drawn double-headed because the direction of flux follows the sign of the local water-potential gradient and is not fixed. Arrow conventions follow the legend. Solid arrows mark material flux: blue for water and purple for Ca^2+^. Dashed arrows mark regulation: green for input coupling or an indirect effect, blue for post-translational regulation. A solid green arrow links a module to an associated regulatory process, and a grey double-headed arrow denotes reciprocal interaction. The single block arrow leaving the cell on the right indicates the overall passage from cellular decoding to the early decoding outputs and does not represent a specific molecular flux. These conventions apply to this figure only, since the other figures use their own legends. The three categories are not mutually exclusive, and the evidence supporting each module differs in strength. The figure shows the spatial and topological organization of the proposed architecture within a cell, whereas Table 1 grades the evidence for each module against three separate criteria. The two are complementary rather than overlapping. Neither should be read as showing that the modules have been demonstrated to act together during a single physiological event.

The second clarification concerns TPC1. It is well established that TPC1 encodes the slow vacuolar channel [65], and that it contributes to systemic Ca^2+^ wave propagation in the salt–stress paradigm [22,25]. The strength of that contribution is nevertheless context-dependent and remains under discussion. Reported phenotypes differ between loss-of-function and gain-of-function alleles, the requirement for TPC1 is not uniform across stimuli or tissues, and the extent to which vacuolar Ca^2+^ release amplifies rather than initiates the wave is not settled. The role assigned to TPC1 here is accordingly that of a context-dependent amplifier and relay within a cell-to-cell propagating system, not that of an obligatory step in every systemic hydraulic response. Taken together, the current evidence argues against a single hydraulic receptor and is consistent with four functionally distinguishable layers, which we treat as a working model rather than as a demonstrated architecture. Plasma-membrane channels provide rapid transduction; wall-associated systems establish mechanical context; aquaporins regulate conductance and signal gain; and vacuolar or cytoplasmic processes provide buffering and protective execution. Table 1 separates evidence for molecular function from evidence for whole-plant hydraulic decoding, and Figure 2 summarizes the layered architecture.

**Table 1:** Candidate modules involved in hydraulic sensing and decoding. Evidence is graded separately against three non-equivalent criteria, because a module may be well supported on one and unsupported on another. E1, direct demonstration that the molecule responds to a mechanical or osmotic variable, typically by electrophysiology in a heterologous system, in reconstituted membranes or by structural determination of a gating transition. E2, demonstration in planta that loss or gain of function alters a local physiological response to a mechanical or osmotic stimulus. E3, demonstration that the module is required for, or quantitatively shapes, a systemic response to a defined hydraulic perturbation delivered without confounding chemical damage. Ratings are assigned as follows: Strong, at least one direct experiment with an appropriate genetic or pharmacological control and independent replication or corroboration; Moderate, supporting evidence that is indirect, obtained in a single system, or open to an alternative interpretation; Limited, suggestive or inferential evidence only. Not applicable indicates that the criterion is not meaningful for that class of module. The three columns must not be read as interchangeable: strong E1 evidence does not license an inference about E3, and the frequent absence of E3 ratings above Moderate is itself one of the principal conclusions of this review.

Module/Layer	Likely Physical Input	Principal Location	Most Defensible Role	Representative Evidence	E1 Channel-Level Mechanosensitivity	E2 Local Function in Planta	E3 Systemic Hydraulic Decoding
OSCA/TMEM63	Membrane tension; osmotic up- or downshift	Plasma membrane	Rapid conversion of membrane mechanics or osmotic state into ion flux and Ca^2+^ signaling	Osmosensitive Ca^2+^-permeable channels [11]; OSCA1 required for hyperosmotic Ca^2+^ rise [12]; mechanically activated gating [13]; OSCA2.1/2.2 are pollen hypo-osmosensors [14]	Strong	Moderate	Limited
MSL channels	Membrane stretch; hypo-osmotic swelling	Plasma and organellar membranes	Tension-gated ion release, osmotic safety, and proposed coincidence gating together with GLRs	MSL9/MSL10 account for root mechanosensitive activity [42]; MSL8 governs pollen hydration [43]; open and closed structures define the gating transition [40]; *msl10* shortens wound-induced slow wave potentials in distal leaves and is epistatic with GLR3.3/3.6 [34]	Strong	Strong	Moderate
MCA proteins	Cell-surface mechanical strain	Plasma membrane	Mechanically induced Ca^2+^ influx and local touch responses	MCA1 required for Ca^2+^ influx and root touch sensing [44]	Moderate	Moderate	Limited
PIEZO	Root-tip compression and tissue deformation	Root-cap plasma membrane	Root mechanotransduction and mechanically evoked Ca^2+^ transients	PZO1 required for root penetration and mechanical Ca^2+^ responses [45]	Moderate	Moderate	Limited; hydraulic role untested
GLR channels	Long-distance turgor change combined with apoplastic L-glutamate	Plasma membrane; phloem sieve elements and xylem contact cells	Conversion of a propagated pressure change into local Ca^2+^ elevation, proposed to be coincidence-gated	GLR3.3/3.6 required for leaf-to-leaf wound signaling [32,33]; two spatially separate cell pools are both required; turgor change activates GLRs locally whereas local glutamate release alone is insufficient [8]	Moderate	Strong	Strong; currently the best-resolved case
Cell-wall integrity modules (FER, THE1, LRX)	Wall strain, pectin status, turgor–wall mismatch	Cell wall–plasma membrane interface	Mechanical context setting, Ca^2+^/ROS coupling, wall repair, and control of vacuolar expansion	THE1 responds to compromised cellulose synthesis [47]; FER maintains wall integrity under salt via cell-specific Ca^2+^ [15]; CWI and immune systems cooperate [48]; FER–LRX sets vacuolar expansion [49]	Limited; sensed variable unidentified	Strong	Limited
Aquaporins (PIPs/TIPs)	Water-potential gradients and regulatory state	Plasma membrane and tonoplast	Regulated conductance, gain control, attenuation and feedback	OST1 phosphorylation of PIP2;1 at Ser121 [16]; ROS-driven internalization lowers root conductivity within minutes [17]; H_2_O_2_ conduction into guard cells [56]	Limited; not primary sensors	Strong	Moderate; strong for gain control
Vacuolar/tonoplast processes	Turgor and tonoplast load	Vacuole/tonoplast	Intracellular buffering, Ca^2+^ relay, quality control and membrane protection	TPC1 is the slow vacuolar Ca^2+^-release channel [65] and contributes to systemic Ca^2+^ wave propagation in a context-dependent manner [22,25]; turgor-dependent tonoplast ATG8ylation after wall damage [18]; wall-derived control of vacuolar expansion [49]	Moderate	Strong	Moderate; contested in detail
Cytoskeleton	Wall–membrane strain transmission	Cortical cytoplasm	Force transmission, receptor organization, trafficking and wall remodeling	Wall–membrane–cytoskeleton continuum [46]; microtubules align with principal stress and reorient under imposed compression [50,51]	Not applicable	Strong for mechanical stress	Limited; no hydraulic test
Phosphorylation modules (SnRK2, RAF)	Osmotic and turgor change; downstream of channel and receptor activation	Cytosol; membrane-associated substrates	Rapid covalent adjustment of channel, aquaporin and transcriptional targets on a seconds-to-minutes scale	Subclass I SnRK2s activated by hyperosmotic and saline stress independently of ABA [52]; B4 Raf-like kinases phosphorylate and activate subclass I SnRK2s [53]; OST1 phosphorylates PIP2;1 [16]	Not applicable	Strong	Limited; not tested with defined hydraulic input
Guard-cell integration machinery	Leaf water status plus ABA, Ca^2+^, ROS and epidermal mechanics	Guard cells/epidermis	Multi-input physiological decoder controlling stomatal aperture	Hydraulic and ABA pathways jointly regulate stomata [5]; aquaporin phosphorylation required for ABA closure [16]	Not applicable	Strong	Strong for integration; indirect for primary sensing

## 4. Early Decoding and Network Integration

### 4.1. Ca^2+^ as an Early Translation Layer

Ca^2+^ is the strongest candidate for a broadly shared biochemical language into which mechanical and osmotic perturbations are translated. Mechanosensitive and osmosensitive channels change Ca^2+^ influx directly [12,44], and transient or oscillatory Ca^2+^ increases are among the earliest responses to abiotic and mechanical stimuli [22]. Ca^2+^ signatures vary in amplitude, frequency, duration, subcellular location and cell type, which gives them far greater coding capacity than a generic alarm signal; in germinating pollen, for instance, the frequency of Ca^2+^ spiking rather than its mere presence carries the osmotic information [14].

Within a hydraulic framework, Ca^2+^ acts as both translator and filter. It converts a physical perturbation into a form read by calmodulins, CBL–CIPK modules, calcium-dependent protein kinases and other decoders, while channel distribution and cellular excitability reshape the input. A brief pressure drop, a sustained hyperosmotic challenge and a repeated pulsed perturbation need not generate equivalent Ca^2+^ dynamics. Demonstrating stimulus-specific Ca^2+^ signatures will require calibrated physical inputs rather than comparison of unrelated stress treatments.

Systemic-signaling studies support hydraulic–Ca^2+^ coupling but rarely establish a universal causal order. In potato, salt-induced hydraulic and Ca^2+^ waves both reach the shoot before photosynthetic activity declines, and blocking Ca^2+^ influx attenuates the earliest photosynthetic phase, which places the ionic event on the causal path without demonstrating that it precedes the physical one [9]. Hydromechanical change can activate Ca^2+^ influx; Ca^2+^-dependent transport and metabolism can in turn alter water movement; and both can be triggered by the same local event. Hydraulic signaling is therefore best regarded as one route into a broader Ca^2+^-encoded network rather than as a self-contained pathway [10].

Decoding also depends on where Ca^2+^ is released. Plasma-membrane influx, vacuolar release through TPC1 [25], chloroplast and mitochondrial signals, and apoplastic Ca^2+^ dynamics can activate different targets even when bulk cytosolic peaks appear similar. Spatially averaged measurements therefore conceal the cellular code most relevant to hydraulic inputs. Reporters targeted to specific organelles and cell layers, combined with simultaneous deformation or pressure measurement, are needed to determine whether distinct hydraulic waveforms generate reproducible Ca^2+^ signatures.

### 4.2. Reciprocal Coupling Among Ca^2+^, ROS and Electrical Activity

ROS are central to rapid systemic signaling through RBOH-dependent production, apoplastic redox relays and redox regulation of channels, aquaporins, kinases and wall enzymes. The coupling is quantitative and mutual: modeling combined with direct imaging showed that Ca^2+^-induced Ca^2+^ release alone cannot account for the observed velocity of the salt-triggered Ca^2+^ wave, and that propagation requires the AtRBOHD NADPH oxidase acting together with the vacuolar channel TPC1 [25]. A hydraulic perturbation can thus promote Ca^2+^ influx; Ca^2+^ can activate RBOHs; and ROS can feed back on channels, membranes and water permeability, with aquaporin internalization providing one documented feedback route [17]. This reciprocal coupling can amplify a local event, extend its spatial reach, or stabilize it long enough to influence physiology [66].

Electrical signals form another rapidly coupled layer. Variation potentials and slow wave potentials propagate through vascular-associated tissues and affect ion transport, photosynthesis, hormones and stomata [26,67]. Their relationship to hydraulics is not merely correlative: the pea pressure-step experiment described in Section 2.2 shows that a hydraulic input is by itself sufficient to elicit slow wave potentials, with electrical amplitude decaying in parallel with hydraulic amplitude along the axis [7]. Conversely, electrical depolarization alters ion and water movement [2]. In many contexts both components arise from a common osmotic or wound event and then reinforce or constrain one another.

The apparent temporal order among hydraulic, Ca^2+^, ROS and electrical components is consequently stimulus- and tissue-dependent. A fixed sequence running from hydraulic input to Ca^2+^, then ROS, then voltage would overstate the evidence. The defensible model is a reciprocally coupled network in which the initiating event biases which component is detected first, while feedback determines duration, propagation and output. Signal termination belongs to the same logic: Ca^2+^/calmodulin-dependent desensitization of GLR3.3 limits systemic wound signaling and helps reset excitability [68].

Wounding demonstrates coincidence detection particularly clearly. Pressure disruption occurs together with cell rupture, glutamate release, electrical depolarization, Ca^2+^ elevation, ROS production and jasmonate signaling. Leaf-to-leaf wound signaling requires glutamate receptor-like genes [33]; glutamate itself acts as a wound signal that triggers long-distance Ca^2+^ waves [32]; and chloride, glutathione-related compounds and insect-derived elicitors introduced directly into the xylem elicit electrical responses without mechanical damage [69]. The last observation is decisive for the present argument: because chemical delivery alone reproduces the electrical response, a physical disturbance is not what encodes wound identity. Robust defense emerges from co-occurring physical and chemical inputs rather than from a single universal alarm channel.

### 4.3. Hormonal Embedding, Peptides and Temporal Layering

The historical contrast between hydraulic and ABA-mediated root-to-shoot signaling is too binary [70,71]. Reciprocal grafting between wild-type and ABA-deficient genotypes showed that root-generated ABA is not required as the long-distance messenger, whereas hydraulic transmission together with ABA biosynthesis and signaling in the shoot is required for the full stomatal response [5]. In soil drought-stressed tomato, hydraulic and endogenous ABA signals make overlapping but non-identical contributions to reductions in stomatal and mesophyll conductance [72].

ABA should therefore be viewed neither as a universal alternative to hydraulic signaling nor as a merely slower downstream output. Local ABA biosynthesis, xylem transport, tissue sensitivity and ABA-dependent regulation of aquaporins and ion channels convert transient hydraulic perturbations into sustained, coordinated responses; the OST1-dependent phosphorylation of PIP2;1 required for stomatal closure is a concrete instance of that conversion [16]. Conversely, altered conductance changes subsequent hydraulic inputs [62]. This reciprocal hormonal embedding explains why immediate responses can precede measurable hormone redistribution while durable acclimation remains hormone-dependent.

Peptides and mobile metabolites add specificity. Root- and shoot-derived peptides influence stomata, vascular development and tissue remodeling [73], while wound-associated molecules identify tissue damage more precisely than pressure loss alone [69]. Cytokinins, ethylene, jasmonates, strigolactones, auxin and brassinosteroids further alter growth, root architecture, wall mechanics and stomatal competence. Their role is not to replace hydraulic cues but to determine which developmental or defense program is engaged.

**Figure 3:**
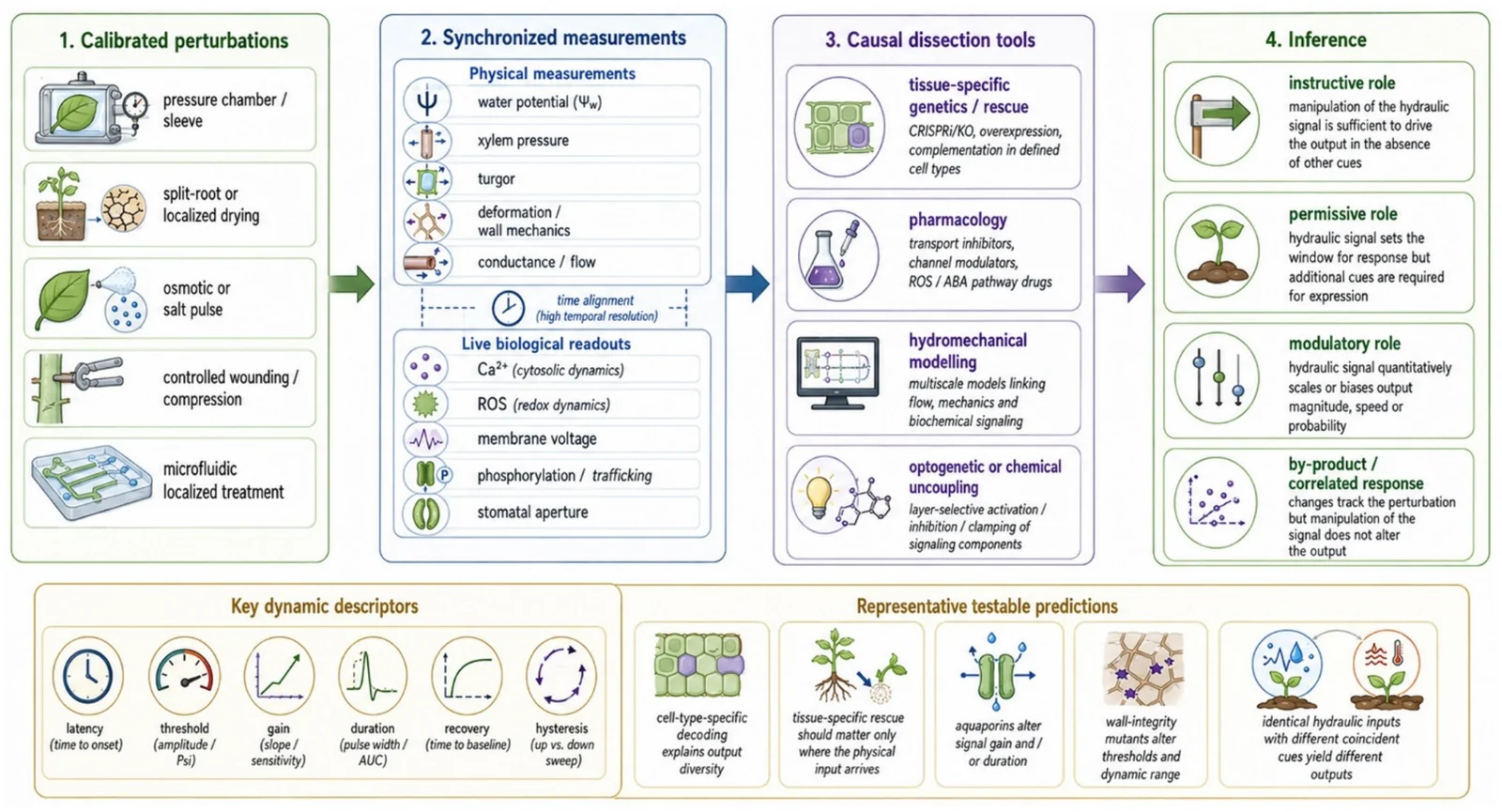
Experimental strategy for testing whether a hydraulic perturbation is decoded as a signal. (1) Calibrated perturbations of defined amplitude, onset, duration and location are imposed using pressure chambers or sleeves, split-root or localized drying systems, osmotic or salt pulses, controlled wounding or compression, and microfluidic localized treatment. (2) Physical and biological layers are measured in the same preparation and aligned on a common clock at high temporal resolution. Physical measurements comprise water potential (Ψ_w_), xylem pressure, turgor, deformation and wall mechanics, and conductance or flow. Live biological read-outs comprise cytosolic Ca^2+^ dynamics, redox dynamics of reactive oxygen species (ROS), membrane voltage, protein phosphorylation and trafficking, and stomatal aperture. (3) Causal dissection combines tissue-specific genetics and rescue, pharmacology, hydromechanical modeling, and optogenetic or chemical uncoupling that selectively activates, inhibits or clamps individual signaling layers. (4) These data discriminate among four inferences. An instructive role means that manipulating the hydraulic signal is sufficient to drive the output when no other cue is present. A permissive role means that the hydraulic signal opens the window for a response but that additional cues are still required. A modulatory role means that it scales or biases the magnitude, speed or probability of an output that would occur anyway. A by-product or correlated response means that changes track the perturbation but that manipulating the signal leaves the output unaltered. Key dynamic descriptors (lower left) define the input–output relationship, and representative testable predictions (lower right) follow from the distributed decoding model. CRISPRi, CRISPR interference; KO, knockout; AUC, area under the curve.

Temporal layering is important. Channel gating, aquaporin regulation, phosphorylation, ROS production and membrane-potential changes occur within seconds to minutes, before large transcriptional shifts [17,25]. Transcription, protein turnover and metabolic reprogramming then consolidate the response over longer periods [74]. Specificity is therefore an emergent network property shaped by the timing, location and combination of physical, ionic, redox, electrical, hormonal and peptide inputs (Table 2; Figure 3).

**Table 2:** Hydraulic signals in relation to other rapid and sustained signaling layers, including reported propagation velocities on a common axis. Onset times and velocities are approximate and depend strongly on species, tissue, stimulus intensity and measurement method; values are given as orders of magnitude rather than as constants, and should not be compared across studies without reference to the original measurement conditions. Phosphorylation is included as a signaling class in its own right, because it operates on the same time scale as the ionic and electrical layers and is not confined to hormone-dependent pathways. It is not propagative, so its velocity entry records the absence of long-distance transmission rather than a slow one.

Signal Class	Primary Basis	Typical Onset/Route	Reported Propagation Velocity	Principal Contribution	Key Limitation	References
Hydraulic/hydromechanical	Water potential, pressure, turgor, deformation and flow	Seconds to minutes; vascular and extravascular continuum	Order of 10 cm s^−1^ (wound-induced front, wheat); attenuates with distance	Rapid physical context; magnitude, rate and route information	No single variable uniquely identifies a stress; sensed variable often unresolved	[2,3,7,23]
Ca^2+^	Channel-mediated cytosolic and organellar Ca^2+^ dynamics	Seconds to minutes; cell-to-cell and vascular-associated	Order of 10^2^ µm s^−1^ (salt-triggered root-to-shoot wave)	Early translation with high coding capacity	Upstream trigger often ambiguous; transfer function from hydraulic input not established	[14,22,25]
ROS	RBOH-dependent production and redox relay	Seconds to minutes; local and systemic	About 8.4 cm min^−1^ (RBOHD-dependent systemic wave)	Amplification, feedback, wall modification and persistence	Can be damaging; rarely stimulus-specific alone	[17,24,25]
Electrical	Membrane-potential and ion-flux changes	Seconds; excitable and vascular-associated tissues	Action potentials of order mm to cm s^−1^; variation potentials slower and decremental	Rapid coordination; coupling to transport and metabolism	Waveform meaning depends on tissue and accompanying signals	[7,26,27]
Phosphorylation/post-translational	Kinase–phosphatase cascades acting on channels, aquaporins and transcription factors	Seconds to minutes; cell-autonomous; ABA-dependent and ABA-independent	Not propagative; acts locally wherever the upstream cue arrives	Fastest covalent adjustment of transport and gain; sets the state on which later signals act	Rarely resolved in time against a defined physical input; branches often not separated	[16,52,53]
Hormonal (ABA-centred)	Biosynthesis, transport, perception and local sensitivity	Minutes to hours; local and vascular	Limited by bulk flow in the transpiration stream and by loading and unloading	Sustained coordination, threshold setting and transcriptional consolidation	Often lags the earliest physical event; origin can be difficult to assign	[5,70,71]
Peptide/mobile molecules	Secreted peptides, amino acids and damage-associated metabolites	Minutes to hours; apoplast and vasculature	Diffusion- and flow-limited; strongly dependent on route	Stimulus identity and receptor-mediated specificity	Range and timing are highly context-dependent	[69,73]

## 5. Integration Across Tissues and Organs

### 5.1. Roots as Signal Generators and Preprocessors

Roots usually encounter soil drying, osmotic shock or heterogeneous salinity before shoots do. Early hydraulic limitation originates at the root–soil interface, where rhizosphere drying reduces water uptake before bulk soil water is depleted [1], and field-grown plants adjust radial root hydraulic conductivity across depth and time rather than maintaining a fixed uptake profile [75]. Root architecture, rhizosphere contact, endodermal barriers, suberization, aquaporin composition, xylem loading and local metabolism all transform the external disturbance before it enters the long-distance pathway [73]. What the shoot receives is therefore a processed variable, not a direct report of soil water status.

Preprocessing can attenuate routine fluctuation, sharpen an abrupt perturbation, or alter its coupling to ion and hormone signals. A root system with high hydraulic plasticity may buffer shallow, reversible drying while preserving information about a more severe or persistent event. Conversely, structural barriers or low conductance can delay shoot exposure until the stress is advanced. These propositions are testable, yet direct comparisons of exported pressure dynamics and downstream signaling among contrasting root architectures remain rare.

Root tissues may also encode spatial information. Drying restricted to young laterals, mature axes or deep roots will encounter different radial resistances and aquaporin complements before reaching the xylem, and measured radial conductivity varies by roughly an order of magnitude depending on root order, developmental stage and method [28]. Root pressure, mucilage, rhizosphere shrinkage and loss of soil contact further modify the boundary condition [1]. Split-root experiments should therefore report which root orders and depths are treated, not only the fraction of the pot that is dry. Such anatomical detail is essential when comparing genotypes with contrasting root systems.

### 5.2. The Vasculature as Transmission Medium and Active Filter

Vessel diameter, pit membranes, pathway redundancy, embolism behavior, hydraulic capacitance and branch geometry jointly determine propagation velocity, attenuation and dispersion. Radial leakage from conduits into surrounding tissue causes an applied pressure step to decay with distance, so both the hydraulic input and the electrical response it evokes are graded rather than all-or-none along the axis [7]. Living xylem parenchyma and adjacent tissues additionally alter ion composition, respond to wall stress and participate in electrical and ROS signaling, which means that physical transmission and biological filtering occur simultaneously.

Development, anatomy and measurement scale substantially change the apparent behavior of a hydraulic perturbation [28]. Under salinity, hydraulic and electrical components travel within a network that also carries ions and hormones [76]. Under drought, stored water and outside-xylem resistance alter the relationship between xylem tension and leaf response [77]. A mechanistic account must therefore measure both the conduit-scale event and the local state of the living cells that receive it.

Capacitance deserves particular attention because it can delay and reshape a signal without changing steady-state conductance. Water released from elastic tissues temporarily decouples transpiration from root uptake, reducing the amplitude of a rapid xylem-pressure change while extending its duration. Embolism and refilling introduce nonlinearity and hysteresis, and the sequence of stomatal closure, embolism and dehydration differs among species [64]. Prior hydraulic experience can also alter the conduit itself. Controlled cavitation and refilling cycles reduce subsequent cavitation resistance in *Populus angustifolia*, *P. tremuloides* and *Helianthus annuus* stems and in *Aesculus hippocastanum* petioles, while leaving *Acer negundo* and *Alnus incana* unchanged, an asymmetry attributed to degradation of intervessel pit membranes [78]. The signal arriving at a leaf may consequently reflect not only the current root environment but also the recent loading history of stems and petioles, and in some species the transmission medium retains a physical record of what has already passed through it.

### 5.3. Bundle Sheath and Mesophyll as Leaf Hydraulic Gates

Bundle-sheath cells occupy the interface between vascular water delivery and the mesophyll and regulate xylem-to-mesophyll conductance through aquaporins. Xylem-borne ABA reduces bundle-sheath water permeability and leaf hydraulic conductance, identifying this layer as a controllable gate rather than a fixed resistance [58], and manipulating plasma-membrane aquaporins in the bundle sheath alters leaf hydraulics and the water-potential signal experienced by downstream tissue [59]. The regulatory mechanisms deserve to be set out, since this layer is the principal site at which a xylem perturbation is edited before the mesophyll experiences it. Bundle-sheath cells form a continuous parenchymatous sleeve around the vasculature, so essentially all water leaving the xylem for the mesophyll crosses their membranes, and their osmotic water permeability therefore sets leaf hydraulic conductance in series with the xylem itself. Three regulatory inputs converge on that permeability. The first is xylem-borne abscisic acid. It reduces bundle-sheath protoplast water permeability within an hour and lowers leaf hydraulic conductance when supplied through the xylem, but not when applied to the leaf surface [58]. That contrast localizes the control point to cells in direct contact with the vascular apoplast. The second is aquaporin abundance and gating at the bundle-sheath plasma membrane, since manipulating these aquaporins alters leaf hydraulics and shifts the water-potential signal transmitted onward to the mesophyll [59]. The third is the ionic and pH status of the xylem sap itself, which is actively maintained rather than passively inherited. Acidification of the apoplast favors aquaporin opening, whereas alkalinization reduces conductance. The bundle sheath can therefore modulate its own permeability by altering the composition of the fluid it is exposed to. Together these make the bundle sheath a regulated valve with a defined mechanism rather than a fixed resistance. Two consequences follow for hydraulic decoding. Because the valve is upstream of the mesophyll and guard cells, its setting determines the amplitude and rise time of the perturbation those cells actually receive, so the same xylem pressure change can be delivered as a steep or a damped event depending on bundle-sheath state. And because the valve is itself controlled by a chemical signal traveling in the same xylem stream, hydraulic and chemical information are combined at this interface rather than arriving independently at the target cell. Leaves therefore act on the perturbation before guard cells respond to it.

Mesophyll cells simultaneously experience changes in water potential, wall strain, intercellular CO_2_, photosynthetic redox balance and evaporative demand. A vascular perturbation is thus transformed into a local combination of hydraulic, metabolic and redox inputs before it reaches guard-cell output. Species and genotypes differ in bundle-sheath conductance, outside-xylem vulnerability, and leaf capacitance, and outside-xylem vulnerability rather than xylem embolism drives much of the leaf hydraulic decline during dehydration [77]. In rice, leaf hydraulic vulnerability triggers the decline in stomatal and mesophyll conductance during drought [79]. These anatomical differences provide a plausible basis for different decoding thresholds.

Bundle-sheath control also creates a route for tissue-specific feedback. ABA or redox regulation of bundle-sheath aquaporins can reduce leaf hydraulic conductance, which may protect the vasculature while intensifying the local water-potential decline in the mesophyll [58,59]. High conductance can instead sustain photosynthesis while increasing demand on the xylem. The adaptive value of either state depends on evaporative demand, soil water supply and the reversibility of the perturbation, which is why a static measurement of leaf conductance cannot describe decoding.

### 5.4. Guard Cells as Multi-Input Decoding Hubs

Guard cells integrate hydraulic state with ABA, Ca^2+^, ROS, ion transport, metabolism and epidermal mechanics. They do not respond in a mechanical vacuum: the bundle-sheath and mesophyll continuum together with the surrounding epidermis determines how a xylem perturbation is translated into guard-cell turgor and aperture [59,80]. Water permeability at the guard-cell membrane is itself part of the decoding step, since ABA-triggered closure requires OST1-dependent phosphorylation of PIP2;1 and the associated rise in osmotic water permeability [16], with aquaporin-facilitated H_2_O_2_ entry linking the same step to redox signaling [56].

Comparative physiology supplies an evolutionary argument for treating hydraulic control as the ancestral layer on which biochemical control was later superimposed. Lycophyte and fern stomata lack the responses to abscisic acid and to epidermal turgor that characterize seed plants, and their behavior is correspondingly predictable from leaf water status alone [81]. This places the transition from passive to active metabolic control after the divergence of ferns, roughly 360 million years ago. In the conifer *Metasequoia glyptostroboides*, daytime closure under moderate water stress is reversed immediately when excised shoots are rehydrated, the signature of a passive hydraulic process rather than a metabolic one [82]. Angiosperm guard cells retain this hydraulic responsiveness and add ABA-dependent control on top of it. Read in this light, the phasing described above is not an experimental artefact but a layered architecture with a phylogenetic history. That history is also why the relative weighting of mechanics and biochemistry should be expected to differ among lineages rather than converge on one canonical scheme.

A useful distinction separates rapid physical permissiveness from sustained biochemical commitment. During fast dehydration or salt shock, altered epidermal and subsidiary-cell water relations can favor pore narrowing before extensive hormonal redistribution; soil hydraulic resistance can regulate stomata directly, without requiring a change in leaf water potential to be the proximate trigger [83]. If the perturbation persists, ABA, Ca^2+^, ROS and metabolic responses stabilize and extend closure [5,72]. The productive question is therefore not whether the response is hydraulic or ABA-mediated, but how mechanics, local water status and biochemical signaling are phased and weighted.

Both excessive and insufficient responsiveness carry costs. Premature closure reduces carbon gain during reversible fluctuations, whereas delayed closure deepens later water deficits. Hydraulic decoding is thus potentially a functional trait defined by response proportionality and timing rather than by high or low conductance. This concept is attractive for crop physiology but still requires direct genetic and field validation [84].

Stomatal kinetics should be evaluated together with reopening. A genotype that closes rapidly but recovers slowly may conserve water during a single event yet lose carbon under repeated short fluctuations. Rapid reopening can be beneficial after transient stress but risky if hydraulic recovery is incomplete. Threshold, closing rate, minimum aperture, reopening lag and hysteresis are therefore distinct components of the input–output relationship and may be controlled by different anatomical and signaling modules [80].

## 6. Comparative Stress Contexts: Drought, Salinity and Wounding

### 6.1. Drought and Transient Soil Drying

As soil water potential declines, resistance at the root–soil interface increases, root and leaf hydraulic conductance can fall, xylem tension rises and leaf turgor changes. Water uptake begins to decline while bulk soil is still comparatively wet, because the limiting resistance develops in the drying rhizosphere rather than in the bulk soil [1]. Dynamic soil hydraulic resistance is itself sufficient to regulate stomata [83], which supports the argument that soil rather than xylem vulnerability sets the early stomatal response [85]. These events precede visible damage and create an early-to-intermediate window in which stomata, growth and root–shoot allocation can still be adjusted.

The temporal sequence is more informative than a hydraulic-versus-chemical dichotomy. Early drought necessarily alters soil–plant hydraulics; ABA biosynthesis, aquaporin regulation, peptides and ROS then reinforce or reshape the response; and severe drought eventually produces hydraulic failure, embolism, metabolic disruption and tissue injury. Comparative work across species shows that stomatal closure, hydraulic decline, embolism and wilting do not occur in a universal order. Stomatal closure and turgor loss are tightly correlated across taxa, yet both decouple from embolism thresholds [86]. The safety margin between closure and embolism varies widely among co-occurring species [87], and the sequence of closure, embolism and aquaporin expression differs even among cultivated species grown side by side [64]. Pragmatic hydraulic theory nonetheless predicts stomatal responses to climatic water deficit with useful accuracy [88], indicating that the variation is structured rather than idiosyncratic.

One experiment remains particularly instructive. Reciprocal grafting between wild-type and ABA-deficient *Arabidopsis* showed that root-generated ABA is not required as the long-distance messenger, whereas hydraulic transmission together with ABA synthesis and signaling in the shoot is required for the full response [5]. Tomato experiments similarly support overlapping hydraulic and endogenous ABA contributions to reduced stomatal and mesophyll conductance [72]. Leaf vulnerability outside the xylem can drive declines in stomatal and mesophyll conductance before extensive embolism occurs [77,79]. These findings place hydraulic and hormonal processes at different, interacting positions rather than making either a universal substitute for the other.

For crops, intermittent and progressive drought probably makes proportional early responses more important than survival of terminal dehydration alone. Relevant traits include root hydraulic plasticity [75], soil–root contact [1], capacitance, outside-xylem conductance [77], aquaporin regulation [57] and stomatal kinetics. ABA-mediated stomatal control during terminal drought remains important in wheat [89], but constitutively stronger closure is not automatically advantageous, because yield depends on the timing of water use as much as on its total [84].

Drought experiments should also distinguish declining soil water potential from increasing soil hydraulic resistance. The same bulk soil water content imposes different root boundary conditions depending on texture, rhizosphere structure and root density [1]. Vapor-pressure deficit can generate a shoot-first hydraulic perturbation even when soil remains wet. Comparing soil-driven and atmosphere-driven events within the same genotype would reveal whether plants decode the origin and rate of a perturbation or only the local water status that the leaf ultimately experiences.

### 6.2. Salinity and Osmotic Shock

Salinity provides a useful separation between an early osmotic phase and a later ionic phase. Immediately after roots encounter salt, external water potential declines and water uptake is inhibited before large quantities of Na^+^ or Cl^−^ accumulate in the shoot. The evidence for this ordering is direct: in maize, 100 mM NaCl produces an exponential drop in root xylem pressure and rapid depolarization of the trans-root potential within about one minute, at which point Na^+^ is not yet detectable in the xylem vessels [2]. In *Arabidopsis*, the same treatment initiates a root-to-shoot Ca^2+^ wave [22], whose RBOHD- and TPC1-dependent propagation is examined in Section 4.2 [25]. In potato, hydraulic and Ca^2+^ waves both reach the shoot before the first measurable decline in photosynthetic activity [9]. In barley leaves, salinity reduces hydraulic conductance, HvPIP2 aquaporin abundance and stomatal conductance in a coordinated manner that exogenous ABA only partially reproduces [60].

The earliest physical perturbation under salt shock can resemble drought, but its onset is generally steeper, and it rapidly coincides with ion-specific and electrical signals. Distal tissues may therefore discriminate salinity from gradual drying through this electro-hydraulic and ionic combination rather than through pressure amplitude alone [26,76]. FER-dependent wall-integrity signaling further illustrates that salinity alters extracellular mechanics as well as osmolarity and ion composition, since maintaining wall integrity under salt requires cell-type-specific Ca^2+^ transients downstream of FERONIA [15].

Glycophytes and halophytes may experience similar initial osmotic shocks yet differ in thresholds for growth restraint, ion compartmentation, water conservation and recovery [20]. Comparative analysis should therefore pair calibrated hydraulic inputs with measurements of Na^+^ and Cl^−^, membrane voltage, Ca^2+^ and tissue-specific conductance rather than treating salt tolerance as a single endpoint [76].

Recovery from salinity is equally informative. Removing external salt produces a hypo-osmotic transition that challenges membranes and walls in the direction opposite to salt application. A sensor or aquaporin state optimized for dehydration need not be optimal for rehydration, and the pollen work showing that hypo-osmotic sensing depends on dedicated OSCA2.1/2.2 function indicates that the two directions can indeed be read by different modules [14]. Paired stress and release experiments can therefore test whether the same components encode both directions of water-potential change.

### 6.3. Wounding and Mechanical Damage

Wounding abruptly breaks pressure continuity, damages vessels and living cells, releases metabolites, changes membrane potential and initiates Ca^2+^, ROS, electrical and jasmonate responses. Leaf-to-leaf transmission of the wound signal requires glutamate receptor-like genes [33], and glutamate release triggers long-distance Ca^2+^ waves that activate distal defense [32]. Because these events are nearly simultaneous, wounding is a powerful but heavily confounded system for studying rapid systemic signaling.

Hydromechanical modeling predicts how local rupture generates both local deformation and long-distance pressure redistribution, and specifies the conditions under which each dominates [3]. Pressure loss alone nevertheless does not identify herbivory or tissue damage. Introducing chloride, glutathione-related compounds or insect-derived elicitors directly into the xylem elicits electrical signaling in the absence of mechanical injury [69], which shows that chemical context rather than physical disturbance carries wound identity. Signal termination is equally structured: Ca^2+^/calmodulin-dependent desensitization of GLR3.3 constrains the duration and spatial spread of systemic wound signaling [68]. The wound response therefore exemplifies coincidence detection rather than proving that hydraulic change is the first or sufficient systemic signal.

Experimental wounds differ markedly in depth, vascular intersection, tissue compression and released sap volume. Standardizing wound geometry and measuring local pressure loss would improve comparison among studies. Mechanical controls that deform tissue without rupture, and chemical controls that introduce elicitors without major pressure disruption [69], are particularly valuable for estimating the contribution of each component. Distal responses should be mapped relative to vascular connectivity, because physical and chemical routes may diverge at branch points.

### 6.4. Shared Logic and Context-Specific Outputs

**Figure 4:**
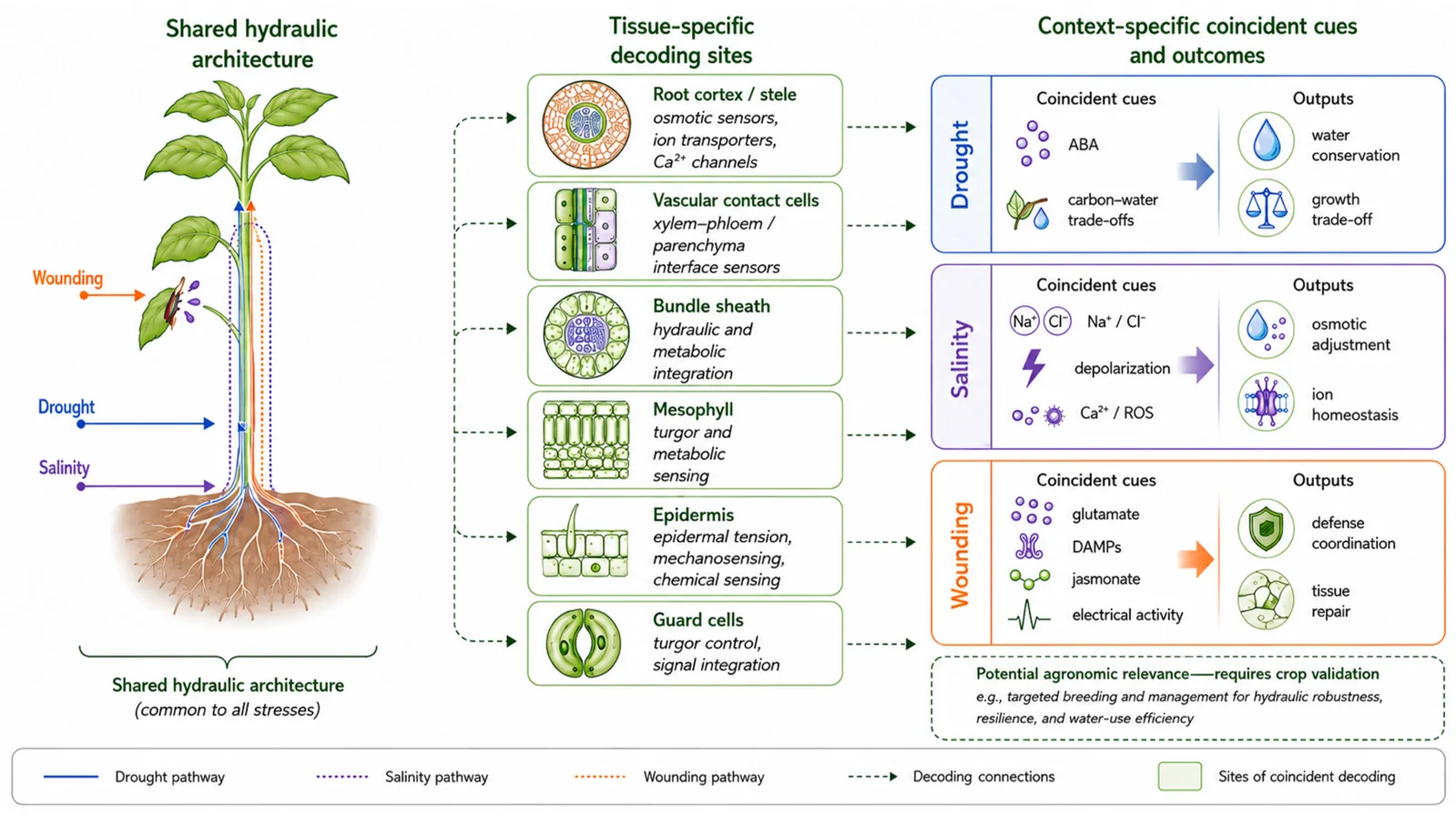
Shared hydraulic architecture with context-specific decoding under drought, salinity and wounding. The figure is arranged in three panels and is deliberately restricted to the elements that are not already presented in Figure 1; the generation of hydraulic perturbations and their propagation and filtering are shown there and are not repeated here. Shared hydraulic architecture (left) shows one vascular system traversed by all three stresses. Colored tracks distinguish the three pathways, and labeled entry points show where each stress is imposed. Drought and salinity both enter at the root–soil interface: drought develops progressively as that interface dries, whereas salinity imposes an acute osmotic shock that lowers root xylem pressure. Wounding enters in the shoot, where local rupture creates a pressure discontinuity. Four properties of this shared system govern what arrives downstream. The vasculature provides long-distance transport, hydraulic capacitance buffers storage and release, attenuation and frequency filtering damp the perturbation, and controlled leakage to the apoplast and symplast determines which tissues are exposed. All four are treated in Section 5 and Section 6, and are represented here by the architecture itself rather than as separate icons. Tissue-specific decoding sites (center) transform the arriving perturbation according to local competence, with root cortex and stele, vascular contact cells, bundle sheath, mesophyll, epidermis and guard cells each combining hydraulic input with different sensors and metabolic context. Physiological identity is assigned at these sites by context-specific coincident cues and outcomes (right): abscisic acid (ABA) and carbon–water trade-offs under drought; Na^+^ and Cl^−^ accumulation with membrane depolarization and Ca^2+^ or reactive oxygen species (ROS) signaling under salinity; and glutamate, damage-associated molecular patterns (DAMPs), jasmonate and electrical activity under wounding. Outputs differ accordingly. The figure illustrates that a shared hydraulic architecture can yield stress-specific outputs. Hydraulic cues supply timing, magnitude and route information, and coincident signals contribute much of the remaining identity. This is not intended to imply that the hydraulic component is informationally inert, since waveform properties themselves differ among the three stresses, as summarized in Table 3. The agronomic panel is drawn with a dashed border to indicate that crop-level validation is still required. Arrow conventions are given in the legend: a solid blue line, a dotted purple line and a dotted orange line trace the drought, salinity and wounding pathways respectively; a solid green arrow denotes information flow; a dashed green arrow denotes a decoding connection; and a pale green fill marks sites of coincident decoding. These conventions apply to this figure only, since Figure 2 uses its own legend for a different set of relationships.

The three stress contexts share a common architecture but differ in temporal profile and accompanying cues. Drought produces a progressive hydraulic trend coupled increasingly to ABA and to carbon–water trade-offs [5,84]. Acute salinity produces a rapid osmotic discontinuity coupled to membrane depolarization and ion identity [2,76]. Wounding produces a pressure discontinuity coupled to damage-associated molecules and defense hormones [32,69]. Hydraulic perturbations are therefore recurring components of stress signaling rather than a universal explanation for systemic responses, and specificity emerges from the joint interpretation of waveform properties, anatomical route, tissue state and coincident signals (Table 3; Figure 4). Table 3 sets out how the hydraulic waveform itself differs among these contexts. Its purpose is two-fold: to answer directly how hydraulic parameters vary with stressor, and to make explicit that the hydraulic component is not a single invariant quantity that can only report severity. The parameters listed are the four dynamic descriptors introduced in Section 2.2, together with direction and anatomical route, and each row is annotated with the highest evidence level that the hydraulic component has actually reached in that context.

## 7. Experimental Strategies and Quantitative Frameworks

### 7.1. Controlled and Spatially Defined Perturbations

Hydraulic signaling is difficult to dissect because physical, osmotic, ionic and hormonal changes are continuous and tightly coupled. Progress requires perturbations with defined amplitude, onset, duration and location, combined with cell-biological read-outs rather than endpoint phenotypes [3]. The most informative historical designs share this property. A calibrated pressure step delivered to an intact root through a pressure chamber isolates the hydraulic variable from wounding and chemical release [7]. A microelectrode combined with a pressure probe resolves the first minute of a salt response in an intact root [2]. Reciprocal grafting separates the site of hormone synthesis from the site of hormone action [5]. Pressure chambers and sleeves, cell pressure probes, split-root systems, localized drying, osmotic pulses, leaf compression and controlled wounding each isolate a different part of the problem [4].

Microfluidic and plant-on-chip systems are particularly useful for imposing localized osmotic change while maintaining imaging access and avoiding some cutting artefacts [90]. Their limitation is scale: a root segment or pollen tube in a device does not reproduce the capacitance, branching and tissue heterogeneity of an intact crop. Microdevice results should therefore be linked to organ- and whole-plant measurements rather than treated as direct surrogates.

### 7.2. Concurrent Measurement of Physical and Biological Layers

A decoding mechanism cannot be inferred from a molecular reporter alone. Physical measurements of water potential, xylem pressure, turgor, deformation, conductance or flow should be temporally aligned with Ca^2+^, ROS, voltage, stomatal, phosphorylation and trafficking read-outs [3]. Studies that combine both layers in one preparation have been the most decisive, whether by pairing xylem pressure with membrane potential in the same root [2] or by pairing an applied pressure step with intracellular electrical recording along a defined axis [7]. Non-contact methods such as multimodal Brillouin microscopy can map mechanical properties and deformation without probe penetration, although calibration against classical hydraulic variables remains essential [91].

Temporal resolution determines causal interpretation. Pressure redistribution and depolarization occur within seconds to approximately a minute [2], whereas ROS propagation, stomatal behavior, transcription and growth diverge over longer windows [25,74]. Experiments should report latency distributions rather than selected time points, and should identify the cell type in which each event begins.

Calibration and uncertainty should be reported explicitly. Pressure probes, psychrometers, optical deformation maps, fluorescence reporters and gas-exchange systems sample different volumes and have different response times. An apparent lead–lag relationship can arise from instrument kinetics or spatial averaging rather than from biology. Synchronizing clocks, measuring sensor response functions and propagating timing uncertainty are therefore necessary whenever differences of only seconds are used to infer causal order.

### 7.3. Separating Hydraulic, Osmotic, Electrical and Chemical Effects

Four design principles are especially useful. First, temporal precedence can exclude messengers that accumulate too late, but precedence alone does not prove sufficiency. Second, spatial separation through split roots, localized dehydration or distant reporters distinguishes local perception from distal response. Third, genetics and pharmacology can place OSCAs, MSLs, MCAs, PIEZO, aquaporins, RBOHs, GLRs, wall-integrity modules and ABA pathways within the network [13,15,25,33,42,45]. Fourth, every molecular perturbation should be paired with physical measurement; otherwise a phenotype may reflect altered signal generation or transport rather than altered perception.

Factorial designs are preferable to sequential single-pathway tests. A channel mutant and an ABA-signaling mutant can be compared under matched slow and fast hydraulic ramps, with and without an ionic cue, so that interaction terms reveal whether the pathways are additive, redundant or conditionally dependent. Rescue restricted to roots, vascular cells, bundle sheath or guard cells can further distinguish the site of perception from the site of physiological execution, an approach already validated for bundle-sheath aquaporins [59]. Such designs are more informative than comparing endpoint survival across unrelated stress severities.

Deliberate uncoupling of downstream layers can test sufficiency. Two channelrhodopsins now permit defined ionic and electrical perturbations in intact plant tissue without mechanical injury [92], and chemical delivery into the xylem provides a complementary route to trigger electrical signaling without pressure disruption [69]. Such experiments can ask which outputs are reproduced by a downstream ionic event and which still require a hydraulic input. True optomechanical control of membrane tension in intact plant tissue remains an important technical goal.

### 7.4. Tissue Resolution and Modeling as Experimental Partners

Cell-type-specific reporters and rescue lines are needed because roots, vascular parenchyma, bundle sheath, mesophyll, epidermis and guard cells do not share the same mechanical boundary conditions. Whole-leaf water potential and transpiration remain indispensable, yet they cannot identify which cell layer assigns physiological meaning to a perturbation. Single-cell and tissue-resolved measurements should therefore be nested within whole-plant hydraulics, and reported alongside the scale at which conductance was determined, since measured values vary systematically with that choice [28].

Hydromechanical models generate falsifiable predictions for propagation speed, attenuation, deformation and tissue exposure [3]. A reporter response occurring faster than a physically plausible pathway requires an alternative mechanism, whereas agreement between predicted and measured spatial patterns strengthens causal inference. Modeling has already produced this kind of discrimination for ionic signaling, where a diffusion-dominated Ca^2+^-induced Ca^2+^ release mechanism was shown to be quantitatively insufficient to explain observed wave velocity, forcing the inclusion of a ROS-assisted step [25]. Future digital-twin approaches may couple poroelastic transport to signaling-network dynamics, but their usefulness will depend on transparent assumptions and independent validation [93]. Table 4 summarizes the complementary experimental strategies.

## 8. Knowledge Gaps, Testable Predictions and Crop Relevance

### 8.1. Bona Fide Sensors and the Physical Variables Sensed

The first unresolved question is whether vegetative tissues possess dedicated hydraulic sensors comparable in mechanistic clarity to the pollen hypo-osmosensors OSCA2.1 and OSCA2.2 [14]. Mechanosensitive channels, wall-integrity pathways and regulated conductance elements are compelling candidates, but their hierarchy is unknown. A universal sensor may not exist, and different tissues may rely on different combinations of membrane mechanics, wall strain, water flux and intracellular buffering.

A second unresolved question is the identity of the direct input in each tissue. Membrane tension, extracellular osmolarity, turgor, wall strain, apoplastic pressure, water flux and cytoplasmic crowding often change together. A convincing mechanism must manipulate the proposed variable independently where possible, demonstrate activation of the candidate module, and connect that activation to a distal physiological response. Poroelastic theory offers the quantitative framework for specifying which variable is actually delivered to a given cell layer [3], and single-channel biophysics offers the complementary test of what that layer can detect [13].

### 8.2. Instructive, Permissive and Modulatory Roles

Hydraulic perturbations need not always be instructive in the strong sense of specifying response identity. In one context a physical event may initiate a response; in another it may be permissive, creating the mechanical conditions under which a hormone or peptide acts; in a third it may modulate threshold, gain or duration. The wound system illustrates the distinction directly, because chemical elicitors delivered without pressure disruption reproduce the electrical output [69] while hydromechanical modeling accounts for the propagation geometry [3]. Experiments should therefore test necessity, sufficiency and interaction rather than asking only whether a pressure change is present.

Stress history can alter the physical baseline from which future inputs are decoded, and one instance of this is already documented at the tissue scale. Cavitation and refilling cycles lower the subsequent cavitation resistance of xylem in several species, an effect attributed to persistent modification of intervessel pit membranes rather than to any change in gene expression [78]. The change is not irreversible: fatigue induced in vivo in sunflower is repaired within the intact plant, so the material property follows a recovery trajectory of its own [94]. Prior hydraulic experience can therefore leave a durable yet reversible mark on a transport structure, which is precisely the form a biophysical memory would take. Whether comparable effects operate on wall cross-linking, tissue elasticity, capacitance, aquaporin phosphorylation, channel abundance or membrane partitioning remains inferential. Such effects should be distinguished from established transcriptional and epigenetic priming, and tested by measuring whether altered material or conductance states persist after molecular stress markers return to baseline.

### 8.3. Testable Predictions

The distributed-decoding model yields seven predictions that are experimentally tractable. First, similar organ-scale pressure changes should generate different local deformations and Ca^2+^ signatures in different cell types. Second, channel mutants should alter decoding only when the relevant physical input reaches the cells that express the channel, which is testable by combining tissue-specific rescue with calibrated perturbation. Third, manipulating aquaporin activity should change response gain and duration even when the primary sensor is intact, a prediction supported by the rapid conductivity changes that follow aquaporin internalization [17]. Fourth, wall-integrity mutants should show altered thresholds when turgor and wall resistance become mismatched [15]. Fifth, identical hydraulic perturbations paired with different ionic or wound-associated cues should produce different physiological outputs [69].

A sixth prediction concerns the quantitative relationship between hydraulic waveform and Ca^2+^ signature, which is currently an explicit gap rather than a contested result. It is widely assumed that Ca^2+^ signatures encode stimulus information, and it is established that hydraulic perturbations elicit Ca^2+^ responses, but no study has systematically varied hydraulic parameters and measured the resulting Ca^2+^ response as a transfer function. The distributed decoding model makes this testable in a specific form. If mechanosensitive channels are the dominant entry route, peak cytosolic Ca^2+^ should scale with the rate of change of the local physical variable rather than with its final amplitude, so that two perturbations reaching the same endpoint by different rise times should produce different peak responses. If vacuolar release provides amplification, the integrated response, measured as area under the curve, should depend on prior loading of internal stores and should show a threshold rather than a graded relation. If the system contains adaptation, a sustained perturbation should produce a transient rather than a sustained Ca^2+^ elevation, and the recovery interval between two pulses should determine the amplitude of the second. Each of these is measurable with existing tools. The design combines a pressure probe or osmotic-pulse system with a ratiometric Ca^2+^ reporter in the same cell, and reports amplitude, rise time, duration, decay constant, and area under the curve for both the physical and the biological trace. Until such transfer functions exist, statements that hydraulic signals are decoded into Ca^2+^ signatures describe a correlation whose form is unknown. A seventh prediction concerns hysteresis. If prior hydraulic experience changes material properties or channel state, the response to an identical second perturbation should depend on the interval and on the recovery trajectory after the first. This can be tested with repeated pressure or osmotic pulses while tracking wall mechanics, aquaporin localization, Ca^2+^ responsiveness and stomatal recovery. Two criteria must be met before such an effect is called memory rather than hysteresis. First, an active biological mechanism must be identified, because history dependence is an ordinary property of viscoelastic and cavitating materials and requires no biology at all. Second, a functional consequence must be demonstrated, because a persistent physical alteration that changes no output is a change in material state rather than a stored signal. Cavitation fatigue satisfies neither criterion at present, and hydraulic hysteresis is used in this review in the material sense unless both are met. Demonstrating a persistent change in the physical input–output curve would provide stronger evidence for hydraulic memory than a later transcriptomic difference alone. Cavitation fatigue and its repair offer a template for the required design, since the persistent change and its recovery were measured on the same material [78,94].

### 8.4. Translational Boundaries and Crop Phenotyping

Hydraulic signaling has crop relevance because field environments fluctuate. Intermittent drought, spatial salinity, rapid changes in vapor-pressure deficit and combined stress expose crops to perturbations that may be reversible if detected and acted upon proportionately. Candidate decoding traits include root hydraulic plasticity [75], tissue-specific aquaporin regulation [95], leaf capacitance, wall mechanics, channel composition and the coupling between hydraulic inputs and Ca^2+^ or electrical excitability. The multi-scale architecture of water transport itself, rather than efficiency at any single scale, is increasingly seen as the relevant design target [93].

Direct crop-level evidence remains limited, so translation should not begin by selecting a single candidate sensor. The priority is dynamic phenotyping: measure the input–response relationship, including latency, threshold, gain, recovery and carbon cost. Controlled perturbations can be combined with gas exchange, thermal imaging, pressure or conductance measurement and yield-relevant recovery assays [84]. Reproductive stages deserve particular attention, because pollen hydration, flowering and grain or fruit set are highly sensitive to water delivery and timing, and pollen is also the tissue in which osmosensing is best resolved mechanistically [14].

Field phenotyping will require proxies that retain mechanistic meaning. Canopy temperature and spectral traits provide high throughput but must be anchored to measured soil and plant water potentials, vapor-pressure deficit and stomatal kinetics. Repeated natural fluctuations may be more informative than a single midday measurement. Genotypes should be compared for the slope and hysteresis of their response curves rather than only for average conductance or final yield. Multi-environment trials are essential, because a rapid conservative response can be beneficial in terminal drought yet costly where stress episodes are frequent and short [84].

A realistic agenda has three steps: define conserved mechanisms in model systems; test homologous modules with tissue-specific manipulation in crops; and validate whether altered decoding improves performance under realistic stress sequences without excessive growth penalties. The target is not maximal conductance or maximal sensitivity, but robust and context-appropriate coordination across anatomy, transport and signaling.

## 9. Conclusions

Hydraulic perturbations are among the earliest physical consequences of drought, salinity and wounding, yet a change in water status becomes a biological signal only when it is perceived and used. The critical conceptual distinction is therefore among hydraulic state, perturbation, propagation, local perception and physiological decoding.

Three conclusions can be drawn with reasonable confidence, each stated at the level of evidence that actually supports it. First, a calibrated hydraulic input is sufficient, on its own, to elicit an electrical response in living cells [7]. This is a Level 3 result obtained under controlled conditions; it removes the possibility that distal electrical responses require damage-released chemistry, but it does not by itself show that hydraulic perturbation is the endogenous carrier under drought, salinity or wounding. Second, the physical event can precede ion delivery and hormone redistribution [2,5], so it is not simply a slow read-out of chemical change. Third, stress identity is not carried by pressure amplitude alone [69]; accompanying chemical and ionic cues contribute substantially, although the hydraulic waveform itself varies in ways that are likely to be informative and that have not been systematically tested.

Beyond these points, the evidence argues against a single universal receptor and is consistent with a distributed architecture, which we advance as a testable model rather than as an established mechanism. Mechanosensitive and osmosensitive channels translate membrane mechanics into ion flux [12,13]; wall-associated systems establish structural context [15]; aquaporins regulate conductance and signal gain [16,17]; and vacuolar processes buffer or execute protective responses [18]. Each component is supported in its own experimental setting, but those settings differ in species, tissue, developmental stage and stimulus, and no study has yet shown the four layers operating together during one physiological event. The architecture should therefore be read as a framework that organizes current evidence and generates falsifiable predictions, not as a mechanism that the evidence already demonstrates. Ca^2+^, ROS, electrical, hormonal and peptide pathways form a reciprocally coupled network that supplies amplification, persistence and stress identity [25].

The next advance will come from causal experiments that measure the physical input and the biological response in the same cells and on the same time scale. Such studies should distinguish instructive, permissive and modulatory roles, and test whether decoding thresholds differ among tissues, genotypes and stress histories. Crop translation is plausible but not yet mature, and should focus on dynamic response proportionality and recovery rather than on a single putative hydraulic sensor.

## Figures and Tables

**Table 3:** Parameters of the hydraulic perturbation under different stressors, and the highest level of evidence reached by the hydraulic component in each context. Evidence levels follow the ladder defined in Section 2.1: Level 1, the perturbation occurs; Level 2, it propagates; Level 3, it is locally transduced; Level 4, it alters physiology with an adaptive consequence. Values are indicative and drawn from the studies cited in Section 2.2 and Section 6; they vary with species, organ, developmental stage and measurement method, and few have been measured within a single comparative experiment. The absence of such a comparative dataset is itself a principal gap identified by this review.

Stressor	Onset Kinetics of the Hydraulic Perturbation	Direction and Amplitude	Persistence and Recovery	Principal Route	Dominant Coincident Cues	Best-Supported Evidence Level
Progressive soil drying	Slow; hours to days, set by rhizosphere and root–soil interface resistance	Sustained decline in water potential and turgor; amplitude accumulates rather than steps	Persistent; recovery depends on rewatering and on embolism repair	Root radial pathways, then xylem; cell-to-cell route favored at low transpiration	ABA accumulation; carbon–water trade-offs	Level 4 for stomatal output; Level 2–3 for the hydraulic component as an instruction
Transient or cyclic water deficit	Intermediate; minutes to hours, often diurnal	Oscillating, partly buffered by capacitance; amplitude frequently below the acclimation threshold	Reversible within a diurnal cycle; hysteresis reported at the tissue scale	Vascular with substantial capacitive exchange	Circadian and metabolic state	Level 1–2; discrimination from informative perturbation not established
Salinity/osmotic shock	Fast; xylem pressure falls within about one minute of exposure	Steep negative step in root xylem pressure; large initial amplitude	Initial osmotic phase gives way to a slower ionic phase over hours	Root radial pathways with rapid transmission to the shoot	Na^+^ and Cl^−^ influx; membrane depolarization; Ca^2+^ and ROS waves	Level 3 established for the early phase; Level 4 for the ionic phase
Wounding/mechanical damage	Fastest; front velocity of the order of 10 cm s^−1^	Abrupt release of xylem tension and local pressure discontinuity; large, spatially graded amplitude	Increase in leaf thickness persists for an hour or longer and recurs after successive wounds	Xylem and adjacent living tissue; strongly dependent on wound geometry	Glutamate and DAMPs; GLR-dependent electrical activity; jasmonate	Level 3 established, including with wounding excluded; Level 4 for defense output
Controlled pressure step (experimental)	Instantaneous by design; rise time set by the operator	Positive or negative as imposed; amplitude decrements with distance because the xylem is hydraulically leaky	Determined by the imposed protocol; ideal for paired-pulse and recovery designs	Xylem, with radial leakage to surrounding cells	None by design; this is the point of the manipulation	Level 3; the reference case for demonstrating sufficiency

**Table 4:** Experimental approaches for testing hydraulic signal generation, propagation, perception and decoding. No single method separates all coupled variables; the strongest inference comes from combining physical measurement, live reporters, genetics and modeling.

Approach	Manipulates or Measures	Scale/Resolution	Main Strength	Main Limitation	Best Causal Use
Pressure chamber/sleeve	Bulk pressure and recovery in leaves, shoots or roots	Organ; seconds to minutes	Reproducible pressure perturbation without wounding [7]	Detached material and limited cellular resolution	Define physiological pressure ranges and rapid outputs
Cell pressure probe/indentation	Turgor, wall stiffness and local compliance	Cell to tissue; seconds to minutes	Direct local physical measurement	Low throughput and difficult access	Link local mechanics to reporter activation
Split-root/localized drying	Spatially restricted drought or salinity	Whole plant; minutes to days	Separates local root input from distal response	Hydraulic and chemical signals still co-occur	Test root-to-shoot transmission and untreated-root responses
Osmotic or salt pulse	Abrupt external water-potential or ionic change	Cell to seedling; seconds to hours	Well-defined onset; compatible with reporters [2,22]	Later osmotic and ionic effects diverge	Resolve early physical versus later ionic phases
Localized wounding/compression	Local rupture, bending or deformation	Local input; systemic read-out	Strong for propagation studies	Damage-associated chemicals confound interpretation	Test coincidence of hydraulic, electrical and chemical signals
Live Ca^2+^/ROS/pH/voltage imaging	Early biological outputs in vivo	Subcellular to organ; seconds to minutes	Temporal and spatial ordering [22,25]	Calibration, motion and tissue penetration	Locate initiation and compare signatures
Genetics/tissue-specific rescue	Candidate sensors, decoders and feedback nodes	Cell to whole plant	Tests necessity and anatomical site [16,59]	Redundancy and developmental compensation	Separate generation, propagation, perception and output
Gas exchange + hydraulics + omics	Conductance, transpiration, protein state and expression	Organ to plant; minutes to days	Connects rapid events to acclimation	Large datasets can obscure causality	Distinguish initiation from consolidation
Microfluidics/plant-on-chip	Localized medium exchange and water-status shifts	Single cell to micro-organ	Precise timing and imaging access [90]	Limited whole-plant realism	Test cell-autonomous sensing and local spread
Optical mechanical mapping	Non-contact stiffness and deformation proxies	Subcellular to tissue	Pairs mechanics with fluorescence [91]	Requires calibration to hydraulic variables	Identify tissues first experiencing deformation
Optogenetic perturbation	Defined ionic or electrical activation	Cell to organ; milliseconds to minutes	Tests downstream sufficiency without wounding [92]	Does not recreate full hydraulic physics	Determine which outputs require hydraulic input

## Data Availability

No new data were created or analyzed in this study. Data sharing is not applicable to this article.
